# Supplementary material for: Unveiling unique microbial nitrogen cycling and nitrification driver in coastal Antarctica
Source: Nat Commun. 2024 Apr 12;15:3143. doi: 10.1038/s41467-024-47392-4 (PMC11014942; doi:10.1038/s41467-024-47392-4)
Supplement: Supplementary file 1 — Supplementary Information [file 41467_2024_47392_MOESM1_ESM.pdf]

Supplementary Information:

Unveiling unique microbial nitrogen cycling and nitrification driver in coastal Antarctica

Authors

Ping Han[1, 2, 3], Xiufeng Tang[1], Hanna Koch[4, 5], Xiyang Dong[6, 7, 8], Lijun Hou[2, 3], Danhe Wang[1], Qian Zhao[1], Zhe Li[1], Min Liu[1, 3*], Sebastian Lücker[4], Guitao Shi[1, 2*]

[1]Key Laboratory of Geographic Information Science (Ministry of Education), School of Geographic Sciences, East China Normal University, 500 Dongchuan Road, Shanghai 200241, China.

[2]State Key Laboratory of Estuarine and Coastal Research, East China Normal University, 500 Dongchuan Road, Shanghai 200241, China.

[3]Institute of Eco-Chongming (IEC), East China Normal University, 3663 North Zhongshan Road, Shanghai 200062, China.

[4]Department of Microbiology, RIBES, Radboud University, Heyendaalseweg 135, 6525 AJ Nijmegen, the Netherlands.

[5]Center for Health & Bioresources, Bioresources Unit, AIT Austrian Institute of Technology GmbH, A-3430 Tulln, Austria.

[6]Key Laboratory of Marine Genetic Resources, Third Institute of Oceanography, Ministry of Natural Resources, Xiamen 361005, China.

[7]State Key Laboratory Breeding Base of Marine Genetic Resources, Xiamen 361005, China.

[8]Fujian Key Laboratory of Marine Genetic Resources, Xiamen 361005, China.

*Corresponding author. Email: gtshi@geo.ecnu.edu.cn (G.S.); mliu@geo.ecnu.edu.cn (M.L.)

Supplementary Results and Discussion

Abundant and diverse comammox *Nitrospira* in coastal Antarctica. Using quantitative PCR of the *amoA* gene, which encodes the alpha subunit of ammonia monooxygenase and serves as a key phylogenetic marker for ammonia oxidizers, we conducted an analysis of the abundance of ammonia-oxidizing archaea (AOA), ammonia-oxidizing bacteria (AOB), and comammox *Nitrospira* in the sediments of six distinct lakes (LA1-LA6) and the proximal soils (LS1-LS6) at these locations (Supplementary Table 1). To distinguish the abundance of clade A and clade B comammox *Nitrospira*, we applied clade-specific *amoA*-targeting primers[1] for qPCR-based analysis. The quantitative results revealed that clade B comammox *Nitrospira* represented the majority of the total ammonia-oxidizing population, indicating their dominance within the ammonia-oxidizing community and surpassing clade A comammox *Nitrospira,* AOA, and AOB (Supplementary Fig. 3). Additionally, we assessed the overall abundance of *Nitrospira* by targeting the *nxrB* gene. This gene encodes the beta subunit of the nitrite oxidoreductase, which is crucial for nitrite oxidation and thus covers both strict nitrite-oxidizing species and comammox *Nitrospira*. The *nxrB* abundances were found to match those of total comammox *Nitrospira amoA* (Supplementary Fig. 3), implying that the majority of *Nitrospira* present in these lake sediments are capable of performing complete nitrification.

Amplicon sequencing of the *amoA* sequences amplified from the six lake sediments

(LA1-LA6) and one proximal soil sample (LS4) in Larsemann Hills area revealed a comparable community structure for AOA, AOB, and comammox *Nitrospira* across all samples. The AOA *amoA* operational taxonomic units (OTUs) detected were predominantly affiliated with the Group I.1b -cluster (Supplementary Fig. 6). The majority of AOB *amoA* OTUs clustered within the genus *Nitrosospira*, but *Nitrosomonas*-like sequences were also obtained from most lake sediments investigated, with the exception of LS6 (Supplementary Fig. 7). Generally, these findings are in alignment with prior studies [2-4]. Surprisingly, clade A comammox *Nitrospira* were not observed in any sample and all *amoA* OTUs obtained were affiliated with clade B (Supplementary Fig. 8).

We observed a significantly greater diversity of comammox *Nitrospira* compared to those of AOA and AOB, as evidenced by the number of *amoA* OTUs identified (Supplementary Table 5). The diversity of all *Nitrospira*, as determined by amplicon sequencing of the *nxrB* gene, was found to be comparable to that of comammox *Nitrospira*. This reinforces the fact that comammox *Nitrospira* dominate in this ecosystem over traditional nitrifiers, including the strict nitrite-oxidizing *Nitrospira* species. All comammox *Nitrospira amoA* OTUs were associated with clade B (Supplementary Fig. 8). Specifically, OTU246, which was dominant in lake LA1, and OTU725, prevalent in most other samples, emerged as the two most abundant comammox *amoA* OTUs. In a similar pattern, two distinct *Nitrospira nxrB* OTUs were observed, with OTU70 being predominantly present in sample LA1, and OTU149 being prevalent in the rest of the samples (Supplementary

Fig. 9), indicating these might stem from the same species. Consistently, the *amoA* and *nxrB* sequences obtained from the two metagenome-assembled genomes (MAGs) of comammox *Nitrospira*, designated La1 and La3, were an exact match to the two dominant *amoA* OTUs (Supplementary Fig. 8) and to the two most abundant *nxrB* OTUs (Supplementary Fig. 9), respectively. This suggests that these particular comammox *Nitrospira* strains are the predominant nitrifiers within these aquatic ecosystems, further substantiating their significance in the nitrification process within this environment.

Regarding the total *Nitrospira* population, members of lineage II, which encompasses both comammox and canonical nitrite-oxidizing species, were preeminent in all lake sediment samples. In stark contrast, lineage IV *Nitrospira*, typically encountered in marine or coastal settings, constituted a significant majority (>75%) of the total *Nitrospira* population in the soil sample LS4 (Supplementary Fig. 9). The prevalence of lineage IV in this context suggests that the surface soil experiences greater influence from sea salt aerosols, derived from the neighbouring Antarctic Ocean (Fig. 1a), than does the lake sediment. Moreover, this observation implies that lineage IV *Nitrospira* may be more adept at thriving in terrestrial habitats[5] than previously recognized. Interestingly, these findings partly contrast a recent study on diverse Antarctic mountainous and glacial soils that identified *Thermoproteota* (AOA) and *Nitrospirota* to be the most abundant ammonia and nitrite oxidizers, respectively[6].

Active nitrifiers revealed by ^{13}C-DNA-SIP. Microcosm incubations with both

unlabelled ($^{12}CO_2$ and $^{14}NH_4Cl$) and labelled ($^{13}CO_2$ and $^{15}NH_4Cl$) substrates (Supplementary Fig. 10) were conducted at temperatures of 4°C and 10°C. We observed NO_3^- accumulation in all samples from LA1 at both temperatures, and in LA2 and LS4 at 10°C (Figure 4a and Supplementary Fig. 11a), indicating active nitrification. Comammox *Nitrospira* were present in all samples, while AOB were identified in some samples only, and AOA were absent (Supplementary Fig. 11b). Specifically, in the LA1 samples, comammox *Nitrospira* were the sole nitrifiers detected in the incubations, while in contrast, both comammox *Nitrospira* and AOB were identified in the LA2 and LS4 samples. Moreover, the prevalence of clade B comammox *Nitrospira* in these samples was confirmed in clade-specific PCR assays, while no clade A comammox species were detected (Supplementary Fig. 11b). In alignment with these *amoA* amplification-based observations, the *nxrB* gene of *Nitrospira* was found in all the samples tested (Supplementary Fig. 11b), confirming the widespread distribution of *Nitrospira* across the different environments sampled.

Subsequent to the DNA-SIP incubations, the abundances of the nitrification functional genes in the different density fractions extracted were analysed by qPCR. This revealed peak shifts in the *amoA* and *nxrB*-based comammox and total *Nitrospira* abundances, respectively, in the ^{13}C-labelled DNA compared to the ^{12}C-DNA for all tested samples (Fig. 4a, Supplementary Fig. 12), confirming the activity of *Nitrospira* and their importance to nitrification and thus NO_3^- production in Antarctic Lake sediments.

Notably, although AOB were detected in both LA2 and LS4 based on AOB-specific *amoA* PCR, their activity was only observed in the incubations of the LS4 samples at 10°C (Supplementary Fig. 4a, Supplementary Fig. 12), further illustrating the differences in nitrifier community composition and their contributions to nitrification between surface soil and lake sediments in Antarctica. According to amplicon sequencing-derived relative *amoA* abundances, the dominant comammox OTUs in the microcosm incubations and the original samples largely were in accordance (Supplementary Fig. 13a), proving that few dominant comammox *Nitrospira* species drive nitrification activity in the soils and lake sediments tested. However, in these mesocosm incubation experiments also new comammox *amoA* OTUs were observed, including few low-abundant clade A comammox *Nitrospira*. The *nxrB* data showed similar concordance between the DNAs extracted from the original samples and ^{13}C-DNA-SIP mesocosm incubations, further supporting the dominance of comammox *Nitrospira* in ammonia and nitrite oxidation (Supplementary Fig. 13b). Notably, lineage IV *Nitrospira* again dominated over lineage II in the ^{13}C-DNA from the LS4 soil incubation. At this site, the ammonia oxidation activity by AOB (Supplementary Fig. 12) might provide additional nitrite, possibly stimulating the activity and growth of nitrite-oxidizing lineage IV *Nitrospira*.

Supplementary Methods

Quantitative PCR (qPCR) analysis. The abundances of functional nitrogen cycling genes *amoA* genes, including nitrogen fixation (*nifH*), nitrification (*amoA, hao*, and

nxrB), denitrification (*napA*, *narG*, *nirS, nirK*, *norB*, and *nosZ*), dissimilatory nitrite reduction to ammonium (DNRA; *nrfA*), assimilatory nitrite reduction (ANR; *nasA* and *nirA*), and anaerobic ammonium oxidation (anammox; *hzo*), were quantified by qPCR on an ABI 7500 Real-Time PCR System (Applied Biosystems, Canada) in 21 μL reaction mixtures. The mixtures contain the following components: 10 μL of Hieff® qPCR SYBR Green Master Mix with Low Rox Plus (Yeasan, China), 0.4 μL of each primer (50 μM), 1 μL of template DNA and 9.2 μL of ddH_2O. The primer sets and amplification conditions are listed in Supplementary Table 2. Standards for quantifying gene abundance were generated from the respective purified PCR products using the QIAquick PCR Purification Kit (Qiagen, Germany) following the manufacturer's protocol. The concentration of the purified PCR products was estimated using Quant-iT PicoGreen dsDNA Assay Kit (ThermoFisher Scientific, China), and converted to gene copy numbers.

PCR, high-throughput sequencing and phylogenetic analysis. The *amoA* genes of AOA, AOB, and comammox *Nitrospira* were amplified using the primer sets CamoA-19F/616R (AOA)[7], amoA1F/2R (AOB)[8], comaA-244f/659r (comammox clade A)[1], comaB-244f/659r (comammox clade B)[1], and Ntsp-amoA-162F/359R (general comammox)[9], respectively. *Nitrospira nxrB* genes were amplified using the primer set nxrB169F/638R[10]. Primer sequences and PCR conditions are listed in Supplementary Table 2. PCR was performed in 50 μL volume system containing 25 μL of 5× Green Taq

Mix (Vazyme, Nanjing, China), 1 μL of each primer (10 μM), 2 μL of template DNA, and 21 μL of ddH$_2$O. PCR products were purified by 1.5% agarose gel electrophoresis and quantified using the Picogreen quantification kit (Invitrogen, Shanghai, China). After the first round of functional gene PCR screening for nitrifiers, we used barcoded primers for subsequent PCR amplification and high-throughput amplicon sequencing. In brief, 10 bp barcodes were added to each forward primer to differentiate the samples. After PCR amplification and product purification, PCR products of the same functional genes, including *amoA* genes of AOA, AOB, and comammox *Nitrospira* (with primer set Ntsp-amoA-162F/359R), and *nxrB* genes of *Nitrospira* were pooled together for subsequent library preparation and paired-end sequenced using an Illumina MiSeq platform (2×300 bp) by Majorbio Bio-Pharm Technology Co. Ltd. (Shanghai, China) according to the manufacturer's protocols.

Raw sequencing reads were processed on the QIIME2 (v.2020-7)[11]. Front-end and reverse-end sequences are spliced together according to the overlap, and low-quality sequences (quality score < 20) and chimeras are removed using the DaDa2 pipeline[12]. The 10-bp barcode sequences were removed and Amplicon Sequence Variants (ASVs) was identified at the 95% similarity level. ASV obtained via QIIME2 analysis were clustered based on 95% similarity into representative OTUs. Maximum likelihood phylogenetic tree was constructed using the OTU sequences based on the best fitting model of IQ-TREE (1000 bootstraps)[13].

The length of the AOA *amoA* amplicon obtained using primers CamoA-19F/616R was longer than the read length from paired-end sequencing (2×300 bp). We speculated that the absence of 30 nucleotides in the middle of the AOA *amoA* fragments would not significantly influence phylogenetic analysis, as evidenced by the phylogenetic comparison of representative AOA *amoA* sequences with and without the 30 nucleotides. Consequently, every single read was first quality-trimmed using fastp[14] with a sliding window of 50 bp. When the average quality score within the window dropped below 30, all subsequent bases were trimmed. Only reads ≥ 260 bp were retained. Then, the non-overlapped paired-end reads generated from the two opposite ends of the same DNA fragment were joined into one sequence and aligned to representative sequences of AOA *amoA* genes from the NCycDB database[15] using hmmalign[16]. In the alignment, the ~30 bp non-hit region at the joint of paired-end reads was trimmed for phylogenetic analysis.

For phylogenetic analyses, the representative sequences of each OTU and reference sequences from each nitrifying guild were aligned using hmmalign[16], and the alignments were trimmed using TrimAL[17] with the "-gappyout" flag. Maximum likelihood trees were constructed using IQ-TREE[13] with the "-MFP -bb 1000" flags for best-fit model selection and 1000 ultrafast bootstraps[18].

Statistical analysis. The number of *amoA* and *nxrB* genes for ammonia oxidizers and *Nitrospira*, respectively, were analyzed using Origin 2018 (OriginLab, USA). Different metrics of alpha diversity (observed OTUs, Chao1, Shannon, Simpson, and phylogenetic

diversity) were computed using QIIME2[11]. Statistical correlations between sediment properties and comammox *Nitrospira amoA* gene abundances were analyzed through canonical correspondence analysis (CCA) using the vegan package of R 3.4.1.

DNA-SIP fractionation. The fractionation of DNA extracted from the SIP incubations was conducted as described previously[19]. Briefly, total DNA of sediment/soil was extracted from 0.5 g sample material using the FastDNA Spin Kit for Soil (MP Biomedicals, Cleveland, OH, USA) according to the manufacturer's instructions. For each treatment, ~3 μg of the extracted DNA was mixed with the CsCl stock solution with an initial density of 1.725 g mL^{-1} in Tris-EDTA (TE) buffer (pH = 8.0). The isopycnic density centrifugation was performed in 5.1 ml Quick-Seal polyallomer ultracentrifugation tube in a VTi65.2 vertical rotor (Beckman Coulter Inc., Palo Alto, CA, USA). The heavy fractions of ^{13}C-labelled DNA were resolved from the light fractions by ultracentrifugation at 177,000 × *g* for 44 h at 20°C. The resulting gradients were fractionated into 15 equal volumes (approximately 340 μL each) by displacing the gradient medium with sterile water from the top of the ultracentrifuge tube using a syringe pump (New Era Pump Systems Inc., Farmingdale, NY, USA), with a precisely controlled flow rate of 0.34 mL min^{-1}. The buoyant density of each fraction was calculated using an AR200 digital hand-held refractometer (Reichert Inc., Buffalo, NY, USA) by measuring the refractive index of 65 μL aliquots. Subsequently, the fractionated DNA was precipitated, washed twice with 70% ethanol, and dissolved in 30 μL TE buffer

as described previously[20]. The abundances of *amoA* of AOB and comammox *Nitrospira* and *nxrB* of all *Nitrospira* were quantified using the primer sets amoA1F/ amoA2R, Ntsp-amoA-162F/359R, nxrB169F/638R (Supplementary Table 2), respectively. For the heavy fractions with the highest amount of comammox *Nitrospira amoA* and *nxrB*, the obtained PCR products were sent for high-throughput amplicon sequencing, with subsequent phylogenetic analysis as described above.

Supplementary Table 1. Details of the sampling locations and geochemical data of surface soil samples, surface sediments and overlying waters from Larsemann Hills (LH) in coastal East Antarctica, as well as surface soil samples from South Victoria Land (SVL).

**Sample type**	**Larsemann Hills**						**South Victoria Land**	
**Soil**	**LS1**	**LS2**	**LS3**	**LS4**	**LS5**	**LS6**	**SVL1**	**SVL2**
Longitude (E)	76°19′26″	76°22′24″	76°22′35″	76°21′53″	76°22'45″	76°22'59″	163°42′32″	163°42′37″
Latitude (S)	69°24′26″	69°23′23″	69°23′10″	69°22′14″	69°23'42″	69°22′32″	74°56′07″	74°56′01″
TOC/%	0.03	0.02	0.02	0.10	0.11	0.09	0.13	0.09
NH_4^+ /μg g^{-1}	4.15	3.61	4.86	0.88	3.52	1.56	0.41	0.83
NO_3^- /μg g^{-1}	2.92	0.67	1.01	0.34	0.25	0.26	2.82	3.62
SiO_3^{2-} /μg g^{-1}	6.87	2.42	0.55	2.44	3.83	3.23	3.26	10.27
PO_4^{3-} /μg g^{-1}	1.09	1.59	0.26	1.69	8.61	0.48	0.44	2.83
$\delta^{15}N$-NO_3^-/‰	0.28	-3.36	-4.94	7.77	-0.31	3.86	-4.52	-1.85
$\delta^{18}O$-NO_3^-/‰	6.20	-5.14	4.48	-7.47	4.94	13.91	1.24	1.76
$\Delta^{17}O$-NO_3^-/‰	n.a.	0.00	0.48	0.40	n.a.	n.a.	0.98	0.72
**Lake sediment**	**LA1**	**LA2**	**LA3**	**LA4**	**LA5**	**LA6**		
Longitude (E)	76°19'26″	76°22'23″	76°22'48″	76°22′12″	76°22'32″	76°22′26″		
Latitude (S)	69°24′26″	69°23'23″	69°23'09″	69°22′23″	69°24'	69°22′28″		
TOC/%	0.07	0.03	0.17	0.07	0.03	0.04		
NH_4^+ /μg g^{-1}	0.65	1.84	5.96	2.64	2.29	1.35		
NO_3^- /μg g^{-1}	0.45	0.16	0.47	0.46	4.9	0.21		
SiO_3^{2-} /μg g^{-1}	1.62	1.07	0.60	1.70	1.74	0.99		
PO_4^{3-} /μg g^{-1}	1.06	0.79	2.18	0.07	1.41	0.11		
$\delta^{15}N$-NO_3^-/‰	-3.4	0.28	-0.39	-3.17	-5.9	2.4		
$\delta^{18}O$-NO_3^-/‰	7.87	7.08	8.5	-5.4	-16.04	25.6		
$\Delta^{17}O$-NO_3^-/‰	1.3	2.01	n.a.	n.a.	0.61	n.a.		
**Lake water**	**LA1**	**LA2**	**LA3**	**LA4**	**LA5**	**LA6**		
Temperature/°C	1.5	1.8	2	1.5	1.2	0.1		
pH	6.32	6.95	7.63	7.66	6.96	7.1		
Salinity/‰	0.06	0.09	0.56	0.52	0.08	0.5		
Conductivity/mS cm^{-1}	0.13	0.18	1.13	1.07	0.17	1.03		
NH_4^+ /μmol L^{-1}	2.43	2.41	3.56	2.93	1.52	1.86		
NO_3^- /μmol L^{-1}	1.17	0.89	0.92	1.01	0.88	0.79		
SiO_3^{2-} /μmol L^{-1}	0.02	2.30	11.51	12.15	2.97	6.96		
PO_4^{3-} /μmol L^{-1}	0.13	0.06	0.13	0.12	0.01	0.03		
$\delta^{15}N$-NO_3^-/‰	4.87	9.62	1.4	6.38	2.78	1.7		
$\delta^{18}O$-NO_3^-/‰	22.27	5.58	11.98	3.75	17.51	28.96		
$\delta^{18}O$-H_2O/‰	-14.62	-14.35	-10.57	-11.8	-12.23	-12.86		
$\delta^{2}H$-H_2O/‰	-104.88	-101.51	-85.21	-92.72	-93.4	-96.67		

Supplementary Table 2. Characteristics of primer sets, including the thermal PCR and qPCR profile, used for the amplification and analysis of functional genes involved in microbial nitrogen cycling processes. These processes include N_2 fixation (*nifH*), nitrification (*amoA, hao*, and *nxrB*), denitrification (*napA*, *narG*, *nirS*, *nirK*, *norB*, and *nosZ*), dissimilatory nitrite reduction to ammonium (DNRA; *nrfA*), assimilatory nitrite reduction (ANR; *nasA* and *nirA*), and anaerobic ammonium oxidation (anammox; *hzo* and *hzs*).

	**Target gene**	**Primer name**	**Forward primer (5'-3')** [a]	**Thermal profile for PCR**	**Thermal profile for qPCR**	**Length (bp)**	**Reference**
**Nitrification**	**AOA-*amoA***	CamoA-19F<br>CamoA-616R	ATGGTCTGGYTWAGACG<br>GCCATCCABCKRTANGTCCA	95 °C for 5 min, followed by 35 cycles of 15 s at 95 °C, 30 s at 53 °C and 45 s at 72 °C.	50 °C for 2 min and 95 °C for 10 min, followed by 40 cycles of 15 s at 95 °C, 30 s at 53 °C and 40 s at 72 °C.	629	21
	**AOB-*amoA***	amoA1F<br>amoA2R	GGGGTTTCTACTGGTGGT<br>CCCCTCTGCAAAGCCTTCTTC	95 °C for 5min, followed by 35 cycles of 15 s at 95 °C, 30 s at 54 °C and 45 s at 72 °C.	50 °C for 2 min and 95 °C for 10 min, followed by 40 cycles of 15 s at 95 °C, 30 s at 54 °C and 40 s at 72 °C.	491	8
	**Comammox-*amoA***	Ntsp-amoA-162F<br>Ntsp-amoA-35R	GGATTTCTGGNTSGATTGGA<br>WAGTTNGACCACCASTACCA	95 °C for 1 min, followed by 35 cycles of 10 s at 95 °C, 40 s at 48 °C and 45 s at 72 °C.	50 °C for 2 min and 95 °C for 10 min, followed by 40 cycles of 15 s at 95 °C, 30 s at 48 °C and 45 s at 72 °C.	198	9
	**Comammox-clade A *amoA***	comaA-244f_a<br>comaA-244f_b<br>comaA-244f_c<br>comaA-244f_d<br>comaA-244f_e<br>comaA-244f_f<br>comaA-659r_a<br>comaA-659r_b<br>comaA-659r_c<br>comaA-659r_d<br>comaA-659r_e<br>comaA-659r_f	TACAACTGGGTGAACTA<br>TATAACTGGGTGAACTA<br>TACAATTGGGTGAACTA<br>TACAACTGGGTCAACTA<br>TACAACTGGGTCAATTA<br>TATAACTGGGTCAATTA<br>AGATCATGGTGCTATG<br>AAATCATGGTGCTATG<br>AGATCATGGTGCTGTG<br>AAATCATGGTGCTGTG<br>AGATCATCGTGCTGTG<br>AAATCATCGTGCTGTG	95 °C for 1 min, followed by 35 cycles of 10 s at 95 °C, 30 s at 53 °C and 45 s at 72 °C.	50 °C for 2 min and 95 °C for 10 min, followed by 40 cycles of 15 s at 95 °C, 30 s at 53 °C and 45 s at 72 °C.	415	1

	**Comammox-clade B *amoA***	comaB-244f_a<br>comaB-244f_b<br>comaB-244f_c<br>comaB-244f_d<br>comaB-244f_e<br>comaB-244f_f<br>comaB-659r_a<br>comaB-659r_b<br>comaB-659r_c<br>comaB-659r_d<br>comaB-659r_e<br>comaB-659r_f	TAYTTCTGGACGTTCTA<br>TAYTTCTGGACATTCTA<br>TACTTCTGGACTTTCTA<br>TAYTTCTGGACGTTTTA<br>TAYTTCTGGACATTTTA<br>TACTTCTGGACCTTCTA<br>ARATCCAGACGGTGTG<br>ARATCCAAACGGTGTG<br>ARATCCAGACAGTGTG<br>ARATCCAAACAGTGTG<br>AGATCCAGACTGTGTG<br>AGATCCAAACAGTGTG	95 °C for 1 min, followed by 35 cycles of 10 s at 95 °C, 30 s at 53 °C and 45 s at 72 °C.	50 °C for 2 min and 95 °C for 10 min, followed by 40 cycles of 15 s at 95 °C, 30 s at 53 °C and 45 s at 72 °C.	415	
	hao	hao1F<br>hao2R	TGAGCCAGTCCAACGTGCAT<br>GCAACAACCCTGCCT CA	95 °C for 1 min, followed by 35 cycles of 30 s at 95 °C, 30 s at 58 °C, 1 min at 72 °C.	50 °C for 2 min and 95 °C for 10 min, followed by 40 cycles of 15 s at 95 °C, 30 s at 58 °C and 1 min at 72 °C.	219	22
	Nitrospira-nxrB	nxrB169F<br>nxrB638R	TACATGTGGTGGAACA<br>CGGTTCTGGTCRATCA	95 °C for 5 min, followed by 35 cycles of 40 s at 95 °C, 40 s at 56.2 °C and 90 s at 72 °C.	50 °C for 2 min and 95 °C for 10 min, followed by 40 cycles of 15 s at 95 °C, 40 s at 56.2 °C and 90 s at 72 °C.	469	23
**Anammox**	***hzo***	hzocl1F1<br>hzocl1R2	TGYAAGACYTGYCAYTGG<br>ACTCCAGATRTGCTGACC	95 °C for 1 min, followed by 35 cycles of 10 s at 95 °C, 30 s at 50 °C, 30 s at 72 °C.	50 °C for 2 min and 95 °C for 10 min, followed by 40 cycles of 15 s at 95 °C, 30 s at 50 °C and 30 s at 72 °C.	994	22
	hzsA	hzsA_526F<br>hzsA_1857R	TAYTTTGAAGGDGACTGG<br>AAABGGYGAATCATARTGGC	95 °C for 1 min, followed by 35 cycles of 30 s at 95 °C, 45 s at 54°C, 1 min at 72 °C.	50 °C for 2 min and 95 °C for 10 min, followed by 40 cycles of 15 s at 95 °C, 45 s at 54 °C and 1 min at 72 °C.	1231	
	hzsB	hzsB_396F<br>hzsB_742R	ARGGHTGGGGHAGYTGGAAG<br>GTYCCHACRTCATGVGTCTG	95 °C for 1 min, followed by 35 cycles of 1 min at 95 °C, 1 min at 59 °C, 45 s at 72 °C.	50 °C for 2 min and 95 °C for 10 min, followed by 40 cycles of 15 s at 95 °C, 1 min at 59 °C and 45 s at 72 °C.	346	24
	hzsC	hzsC745f<br>hzsC862r	CCRAAGAACTGGYTDCCKGTDTG<br>TAHGGATTNCCRTCRTARTTRTT	95 °C for 1 min, followed by 35 cycles of 45 s at 95 °C, 45 s at 55 °C, 50 s at 72 °C.	50 °C for 2 min and 95 °C for 10 min, followed by 40 cycles of 15 s at 95 °C, 45 s at 55 °C and 50 s at 72 °C.	117	
**Assimilatory nitrate**	***nasA***	*nasA*1735	ATNGTRTGCCAYTGRTC	95 °C for 1 min, followed by 35 cycles of 1 min at 93 °C, 20 s at	50 °C for 2 min and 95 °C for 10 min, followed by 40 cycles of 15 s at 95 °C,	198	25

reduction		*nasA*1933	CARTGCATNGGNAYRAA	55.5 °C, 1 min at 72 °C.	20 s at 55.5 °C and 1 min at 72 °C.		
	narB	narB430	AACACIACGCTGTGTATGGC	95 °C for 1 min, followed by 35 cycles of 1 min at 93 °C, 1 min at 57 °C, 1 min at 72 °C.	50 °C for 2 min and 95 °C for 10 min, followed by 40 cycles of 1 min at 93 °C, 1 min at 57 °C and 1 min at 72 °C.	589	26
		narB1019	GARTTIGCCTGICCGGTCA				
	nirA	nirA136	TGGTGGGGICTITWCCACCAG	95 °C for 1 min, followed by 35 cycles of.	50 °C for 2 min and 95 °C for 10 min, followed by 40 cycles of 15 s at 95 °C, 45 s at 50 °C and 1 min at 72 °C.	537	26
		nirA673	CGACRCGRACRTTGAABCC				
**DNRA/Denitrification**	***narG***	1960m2f	TAGTGGGCAG GAAAACTG	95 °C for 1 min, followed by 35 cycles of 15 s at 95 °C, 30 s at 63°C, 30 s at 72 °C.	50 °C for 2 min and 95 °C for 10 min, followed by 40 cycles of 15 s at 95 °C, 30 s at 63 °C and 30 s at 72 °C.	90	27
		2050m2r	CGTAGAAGAAGCTGGTGCTGTT				
**DNRA**	***nrfA***	nrfAF1	GCNTGYTGGWSNTGYAA	95 °C for 1 min, followed by 35 cycles of 30 s at 94 °C, 30 s at 45 °C, 1 min at 72 °C.	50 °C for 2 min and 95 °C for 10 min, followed by 40 cycles of 30 s at 94 °C, 30 s at 45 °C and 1 min at 72 °C.	500	28
		nrfAR1	TWNGGCATRTGRCARTC				
**Denitrification**	***nirS***	cd3aF	GTSAACGTSAAGGARACSGG	95 °C for 1 min, followed by 35 cycles of 30 s at 95 °C, 40 s at 58 °C, 1 min at 72 °C.	50 °C for 2 min and 95 °C for 10 min, followed by 40 cycles of 15 s at 95 °C, 40 s at 58 °C and 45 s at 72 °C.	525	29
		R3cd	GASTTCGGRTGSGTCTTGA				
	nirK	nirK876	ATYGGCGGVAYGGCGA	95 °C for 1 min, followed by 35 cycles of 30 s at 95 °C, 15 s at 57 °C, 1 min at 72 °C.	50 °C for 2 min and 95 °C for 10 min, followed by 40 cycles of 15 s at 95 °C, 15 s at 57 °C and 45 s at 72 °C.	164	30
		nirK1040	GCCTCGATCAGRTTRTGGTT				
	nosZ	nosZ2F	CGCRACGGCAASAAGGTSMSSGT	95 °C for 1 min, followed by 35 cycles of 30 s at 95 °C, 40 s at 58 °C, 1 min at 72 °C.	50 °C for 2 min and 95 °C for 10 min, followed by 40 cycles of 15 s at 95 °C, 40 s at 58 °C and 45 s at 72 °C.	267	31
		nosZ2R	CAKRTGCAKSGCRTGGCAGAA				
	norB	norB1f	CGNGARTTYCTSGARCARCC	95 °C for 1 min, followed by 35 cycles of 1 min at 93 °C, 1 min at 55 °C, 1 min at 72 °C.	50 °C for 2 min and 95 °C for 10 min, followed by 40 cycles of 15 s at 95 °C, 1 min at 55 °C and 1 min at 72 °C.	669	27
		norB8r	CRTADGCVCCRWAGAAVGC				
**N_2 fixation**	***nifH***	nifHF	AAAGGYGGWATCGGYAARTCCACCAC	95 °C for 1 min, followed by 35 cycles of 10 s at 95 °C, 30 s at 55 °C, 30 s at 72 °C.	50 °C for 2 min and 95 °C for 10 min, followed by 40 cycles of 15 s at 95 °C, 30 s at 55 °C and 30 s at 72 °C.	342	32
		nifHR	TTGTTSGCSGCRTACATSGCCATCAT				

**Aerobic denitrification**	***napA***	v66	TAYTTYYTNHSNAARATHATGTAYGG	95 °C for 1 min, followed by 35 cycles of 1 min at 94 °C, 1 min at 50 °C, 2 min at 72 °C.	50 °C for 2 min and 95 °C for 10 min, followed by 40 cycles of 15 s at 95 °C, 1 min at 50 °C and 2 min at 72 °C.	707	33
		v67	DATNGGRTGCATYTCNGCCATRTT				

Supplementary Table 3. Features of the retrieved MAGs of ammonia-oxidizing archaea (AOA), complete ammonia oxidizers (comammox), and nitrite-oxidizing bacteria (NOB) of the *Nitrospira* genus, as reconstructed from coastal East Antarctic Lake sediment and soil metagenomes. The table also includes data on their relative abundance in the respective metagenomes. "-", not detected.

**Group**	**MAGs**	**Comple-teness (%)**	**Redun-dancy (%)**	**Genome size (Mb)**	**GC (%)**	**Relative abundance (%) Larsemann Hills** (Sediment/Soil)					
						**LA1/LS1**	**LA2/LS2**	**LA3/LS3**	**LA4/LS4**	**LA5/LS5**	**LA6/LS6**
I.1b-AOA	*Nitrososphaera* sp. La5-A1	98.06	1.94	3.563	37.9	-/-	0.077/-	0.009/-	0.013/-	**0.156**/-	-/-
I.1b-AOA	*Nitrosocosmicus* sp. Ls6-A2	85.36	2.43	1.176	29.1	-/-	0.048/0.008	0.012/0.056	0.021/-	0.049/-	-/**0.076**
I.1b-AOA	*Nitrosocosmicus* sp. La5-A2	82.52	3.4	2.473	29.6	-/-	0.002/-	-/-	-/-	**0.064**/-	-/-
I.1b-AOA	*Nitrosocosmicus* sp. Ls6-A1	75.35	0.97	2.132	33.9	-/-	-/-	-/-	-/-	-/-	-/**0.149**
I.1b-AOA	*Nitrosocosmicus* sp. Ls1-A1	52.71	5.02	1.719	29.6	0.008/**0.132**	0.007/-	-/-	-/-	-/-	-/-
Comammox clade B	*Nitrospira* sp. La1-X1	96.82	2.73	4.043	56.7	**0.173**/0.014	0.005/-	-/-	-/-	0.002/-	-/-
Comammox clade B	*Nitrospira* sp. La3-X1	91.31	4.09	3.574	56.6	-	0.005/-	**0.047**/-	0.028/0.006	-/-	0.027/-
Comammox clade B	*Nitrospira* sp. Ls1-X1	84.44	1.42	3.498	55.3	0.043/**0.151**	0.008/-	-/-	-/-	0.003/-	-/-
Comammox clade B	*Nitrospira* sp. La1-X2	77.67	7.12	5.392	55.2	**0.053**/0.058	0.005/-	-/-	-/-	0.003/-	-/-
NOB lineage IV	*Nitrospira* sp. Ls6-N1	76.06	5.51	2.861	50.2	-/-	-/-	0.002/-	-/0.011	-/0.002	-/**0.108**
NOB lineage IV	*Nitrospira* sp. La5-N1	75.72	2.49	2.703	56.0	-/-	-/-	-/-	-/-	**0.065**/-	-/-
NOB lineage II	*Nitrospira* sp. Ls1-N1	73.63	3.69	2.637	56.2	0.002/**0.043**	0.040/-	0.002/-	-/-	0.014/-	-/-

Supplementary Table 4. Information of reference genomes for comammox *Nitrospira*, AOA and NOB *Nitrospira* phylogenomic and Average Nucleotide Identity (ANI) analysis in this study.

Nitrifier group	Genome	NCBI Accession no.
	Nitrospira sp. Hainich-H41	GCA_919901595.1
	Nitrospira sp. Hainich-H51	GCA_919902055.1
	Nitrospira sp. RSF11	GCA_005116945.1
	Nitrospira sp. RSF3	GCA_005116835.1
	Nitrospira sp. RSF6	GCA_005116885.1
	Nitrospira sp. RSF7	GCA_005116825.1
	Nitrospira sp. WB16	GCA_013140535.1
	Nitrospira sp. ES bin41	GCA_014379455.1
	Nitrospira sp. palsa1310	GCA_003135435.1
	Nitrospira sp. palsa1315	GCA_003152135.1
**Comammox *Nitrospira***	*Nitrospira* sp. LM bin98	SAMN11408089
	Nitrospira sp. RCB	GCA_005239475.1
	Nitrospira sp. CG24C	GCA_002869885.2
	Nitrospira sp. CG24E	GCA_002869895.2
	Nitrospira sp. CG24A	GCA_002869925.2
	Nitrospira sp. GGF bin22	SAMN11408086
	Nitrospira sp. LPPL bin219	SAMN11408092
	Nitrospira sp.THK bin2	SAMN11408107
	Nitrospira sp. HD017	GCA_900236945.1
	Nitrospira sp. Smid bin44	SAMN11408101
	Nitrospira sp. WB04	GCA_013140715.1
	Ca. Nitrosocosmicus oleophilus MY3	GCA_000802205.2
	Ca. Nitrosocosmicus sp. Kfb	GCA_007826885.1
	Ca. Nitrosocosmicus exaquare G61	GCA_001870125.1
**I.1b-AOA**	*Nitrososphaera viennensis* EN76	GCA_000698785.1
	Ca. Nitrososphaera evergladensis SR1	GCA_000730285.1
	Ca. Nitrososphaera gargensis Ga9.2	GCA_000303155.1
	Nitrososphaera-like AFS	GCA 009898475.1
	Ca. Nitrosotalea devanaterra ND1	GCA 900065925.1
**I.1a-associated-AOA**	*Ca.* Nitrosotalea bavarica SbT1	GCA_900167955.1
	Ca. Nitrosotalea sinensis ND2	GCA_900143675.1
	Ca. Nitrosotenuis uzonensis N4	GCA_000723185.1
	Ca. Nitrosotenuis cloacae SAT1	GCA_000955905.3
	Ca. Nitrosotenuis chungbukensis MY2	GCA_000685395.1
	Ca. Nitrosopelagicus brevis CN25	GCA_000812185.1
	Ca. Nitrosarchaeum limnium BG20	GCA_000241145.2
	Ca. Nitrosarchaeum limnium SFB1	GCA 000204585.1
**I.1a-AOA**	*Nitrosarchaeum koreense* MY1	GCA_000220175.2
	Ca. Nitrosopumilus koreense AR1	GCA_000299365.1
	Ca. Nitrosopumilus piranensis D3C	GCA_000875775.1
	Nitrosopumilus maritimus SCM1	GCA_000018465.1
	Ca. Nitrosopumilus adriaticus NF5	GCA_000956175.1
	Ca. Nitrosopumilus salaria BD31	GCA_000242875.3
	Ca. Nitrosopumilus sediminis AR2	GCA_000299395.1
**Th-AOA**	*Ca.* Nitrosocaldus islandicus 3F	GCA_002906215.1
	Ca. Nitrosocaldus cavascurensis SCU2	GCA_900248165.1

Nitrifier group	Genome	NCBI Accession no.
	Nitrospira sp. MC12	GCA_016106465.1
	Nitrospira sp. Bin 54	GCA_009885705.1
	Nitrospira sp. RSF8	GCA_005116815.1
	Nitrospira sp. P-RSF-IL-22	GCA_013141215.1
	Nitrospira sp. UBA5699	GCA_002420105.1
	Nitrospira sp. KM1	GCF_011405515.1
	Nitrospira japonica	GCF_900169565.1
**Lineage II *Nitrospira*-NOB**	Nitrospira sp. CR1.3	GCA_014055525.1
	Nitrospira sp. UBA6493	GCA_002435325.1
	Nitrospira sp. ST-bin5	GCA_002083555.1
	Nitrospira lenta	GCF_900403705.1
	Nitrospira sp. WB-IL-20	GCA_013140455.1
	Nitrospira sp. RSF13	GCA_005116865.1
	Nitrospira sp. CG24D	GCA_002869855.2
	Nitrospira moscoviensis	GCA_001273775.1
	Nitrospira sp. OLB3	GCA_001567445.1
	Nitrospira sp. H3 NOB1	GCA_015709715.1
	Nitrospira sp. RBC073	GCA_902500995.1
**Lineage I *Nitrospira*-NOB**	Nitrospira sp. CR1.1	GCA_014055465.1
	Nitrospira sp. TMP-bin3	GCA_018242665.1
	Nitrospira defluvii	FP929003.1
	Nitrospira sp. CR2.1	GCA_014055475.1
**undefined lineage -NOB**	*Nitrospira* sp. SR bin148	SAMN11408106
	Nitrospira sp. bin75	GCA_002238765.1
**Lineage IV *Nitrospira*-NOB**	*Nitrospira* sp. CO36405bin 1	GCA_011523385.1
	Nitrospira sp. HKST-UBA04	GCA_020430285.1
	Nitrospira sp. RBC003	GCA_902500695.1

Supplementary Table 5. The gene-based diversity indices for of AOA, AOB and comammox *Nitrospira amoA* and *Nitrospira nxrB* genes in LA1-LA6 (lake sediments) and LS4 soil in Larsemann Hills area in East Antarctica. n.a., not available; PD, phylogenetic diversity.

**Sample**	**AOA/AOB-*amoA***					**Comammox *Nitrospira-amoA***					***Nitrospira-nxrB***				
	Observed OTUs	Shannon	Simpson	Chao	PD	Observed OTUs	Shannon	Simpson	Chao	PD	Observed OTUs	Shannon	Simpson	Chao	PD
LA1	3/n.a.	0.1/n.a.	0.0/n.a.	3/n.a.	0.3/n.a.	36	1.6	0.5	41	1.4	12	1.6	0.6	12	0.5
LA2	13/5	2.3/2.3	0.7/0.8	13/15	1.2/0.4	40	2.6	0.8	40	1.6	33	3.2	0.8	33	1.3
LA3	3/5	1.0/2.3	0.4/0.8	3/15	0.3/0.5	36	0.7	0.2	36	1.5	24	1.3	0.4	24	0.9
LA4	6/4	1.4/2.0	0.6/0.8	6/10	0.7/0.5	41	1.5	0.4	41	2.0	24	1.1	0.3	26	1.0
LA5	4/6	1.5/2.6	0.6/0.8	4/21	0.4/0.5	39	2.6	0.8	47	2.0	18	3.0	0.9	19	0.8
LA6	n.a./2	n.a./1.0	n.a./0.5	n.a./3	n.a./0.1	35	1.0	0.2	39	1.4	10	1.3	0.4	10	0.5
LS4	10/17	2.1/4.1	0.7/0.9	10/153	0.9/1.2	31	1.5	0.5	32	1.3	20	1.8	0.6	23	0.7

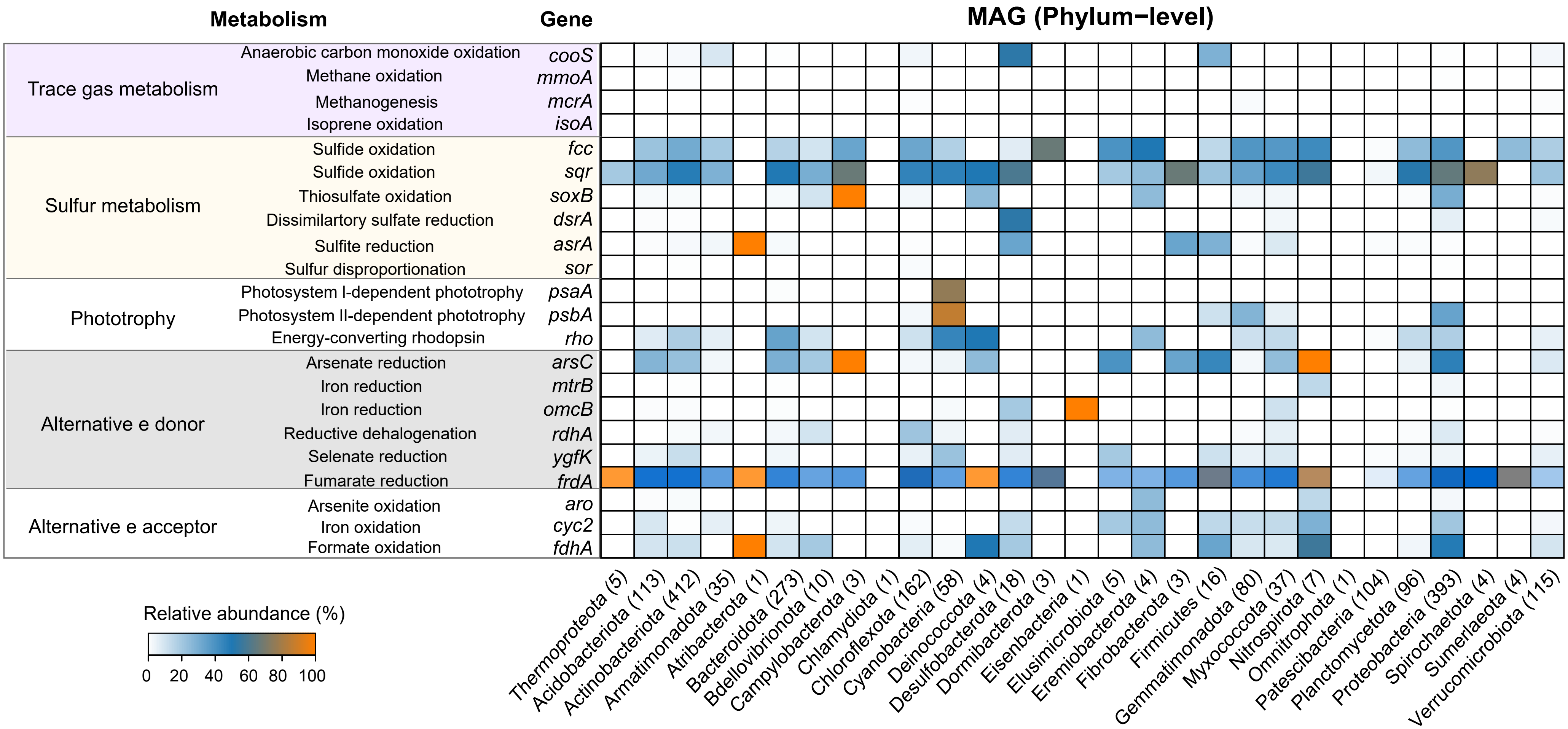

Supplementary Fig. 1. Metabolic gene distribution and identification in the obtained metagenome-assembled genomes (MAGs). Metabolic genes encode enzymes that participate in trace gas metabolism, sulfur metabolism, phototrophy, and the utilisation of alternative electron donors and acceptors. The heatmap on the right reveals the prevalence of these genes across a comprehensive collection of 1968 MAGs, spanning 29 distinct phyla.

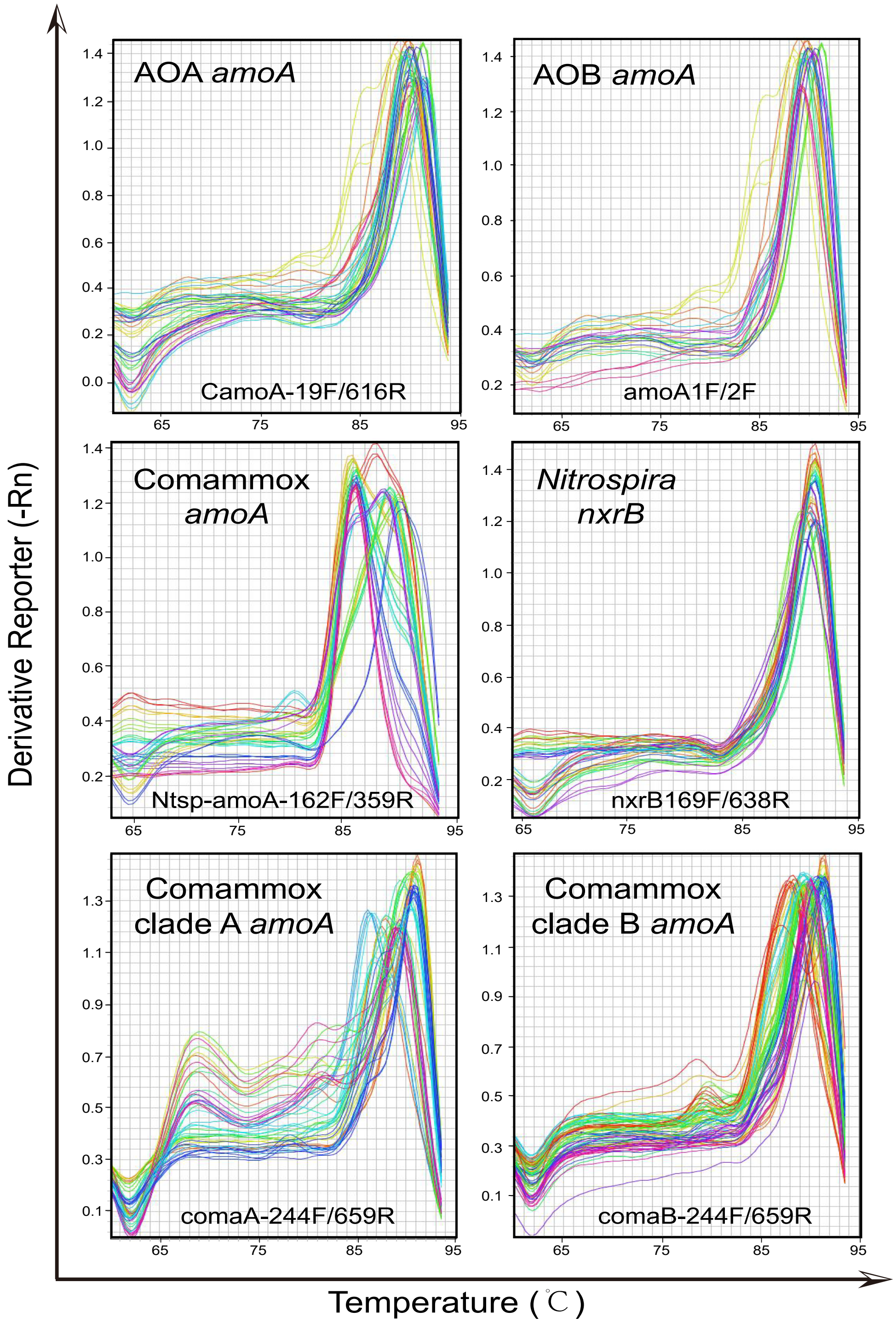

Supplementary Fig. 2. Melting curves demonstrate the specificity of various qPCR primers. The primers target different functional genes and nitrifying groups, including AOA-, AOB-, comammox-*amoA* and *Nitrospira-nxrB*.

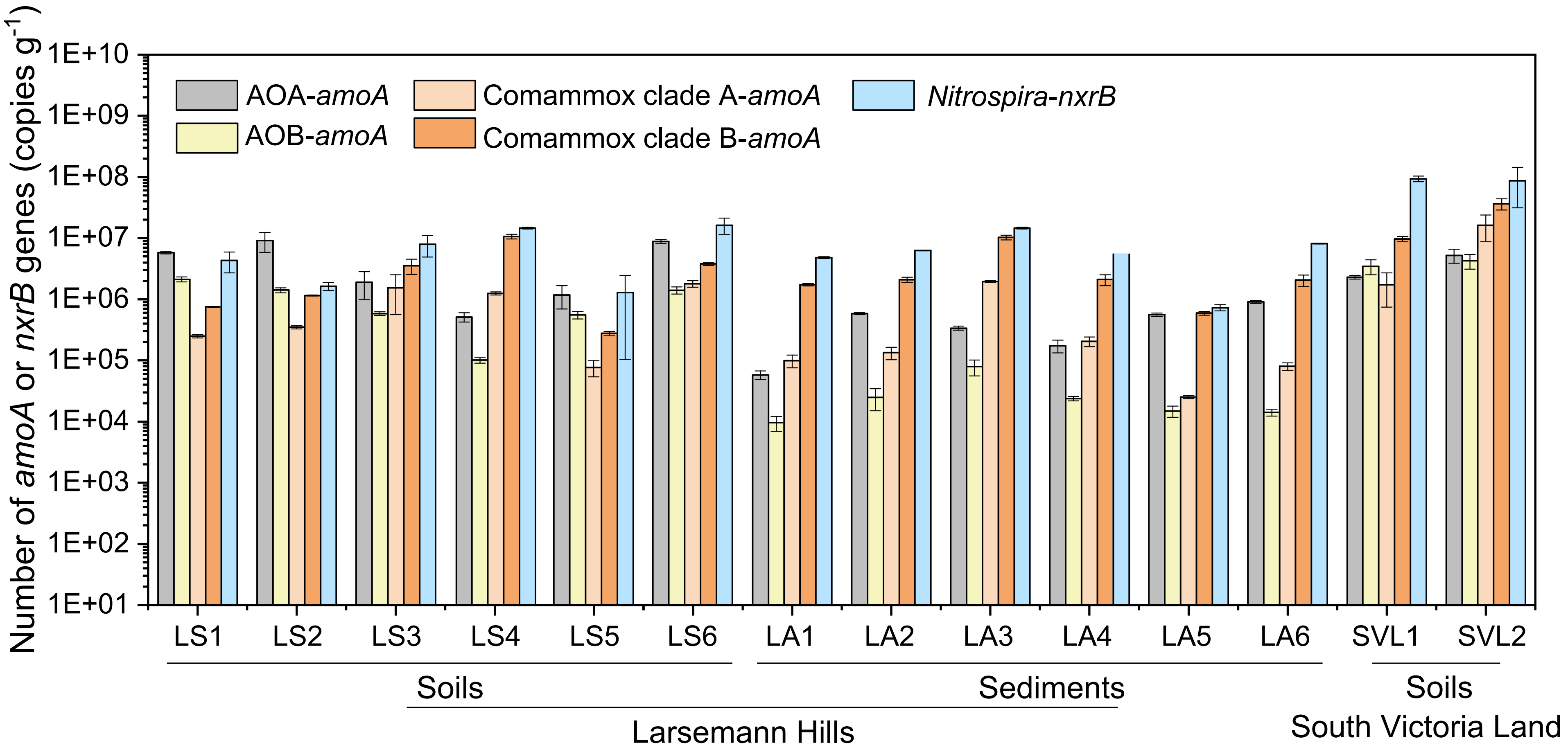

Supplementary Fig. 3. Quantitative PCR-based abundance of key nitrification genes. Quantitative PCR-based abundance of AOA-, AOB-, comammox *Nitrospira-amoA*, and *Nitrospira- nxrB* genes in the samples analysed in this study. Comammox clades A and B were individually quantified using the specific primer sets listed in Supplementary Table 2.

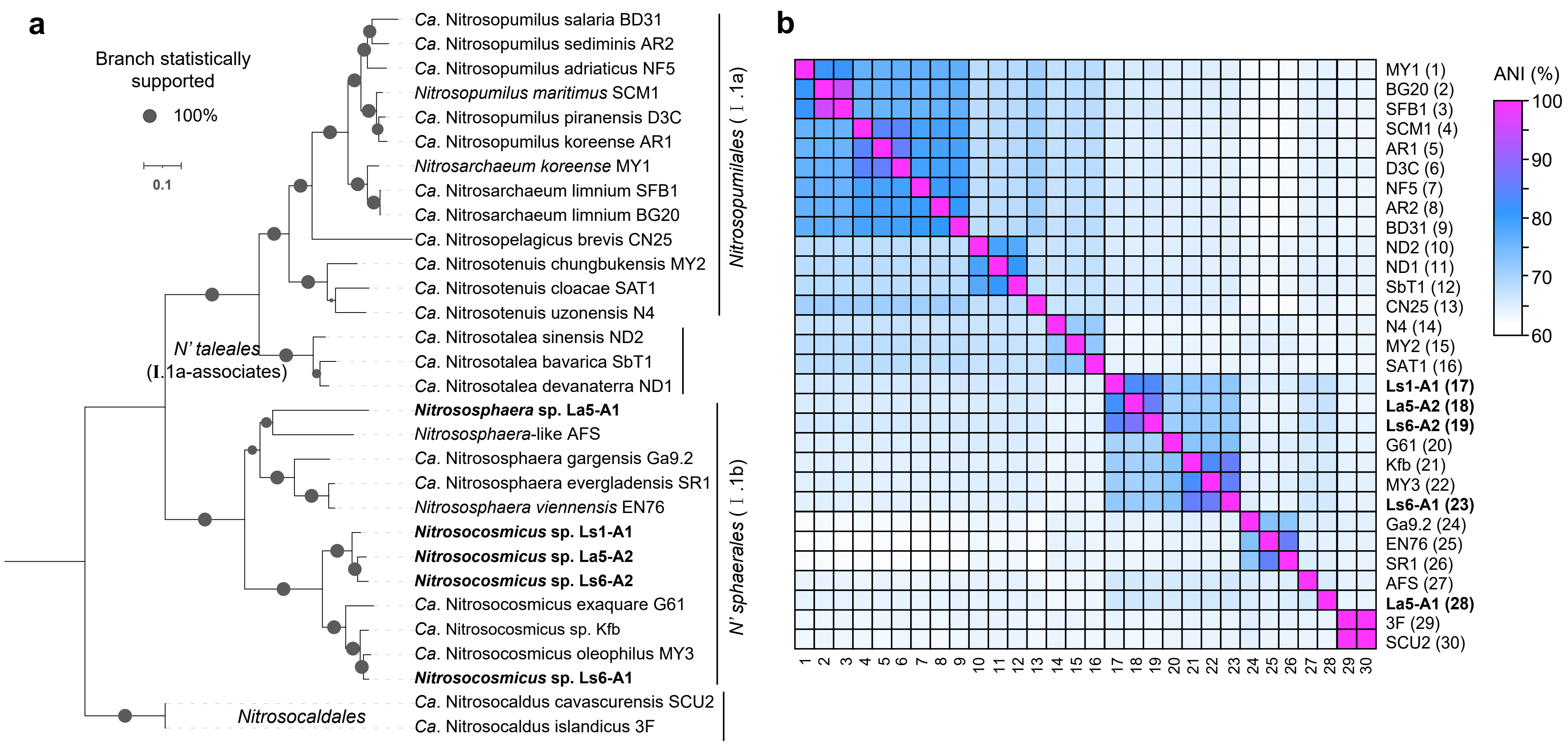

Supplementary Fig. 4. Phylogenomic analysis of AOA. (a) Maximum likelihood phylogenomic tree of AOA MAGs obtained here and selected reference genomes from the NCBI database. **(b)** Average Nucleotide Identity (ANI) analysis; the AOA metagenome-assembled genomes (MAGs) identified in this study are highlighted in bold, AOA reference genomes are indicated by the same strain identifier as given in (a).

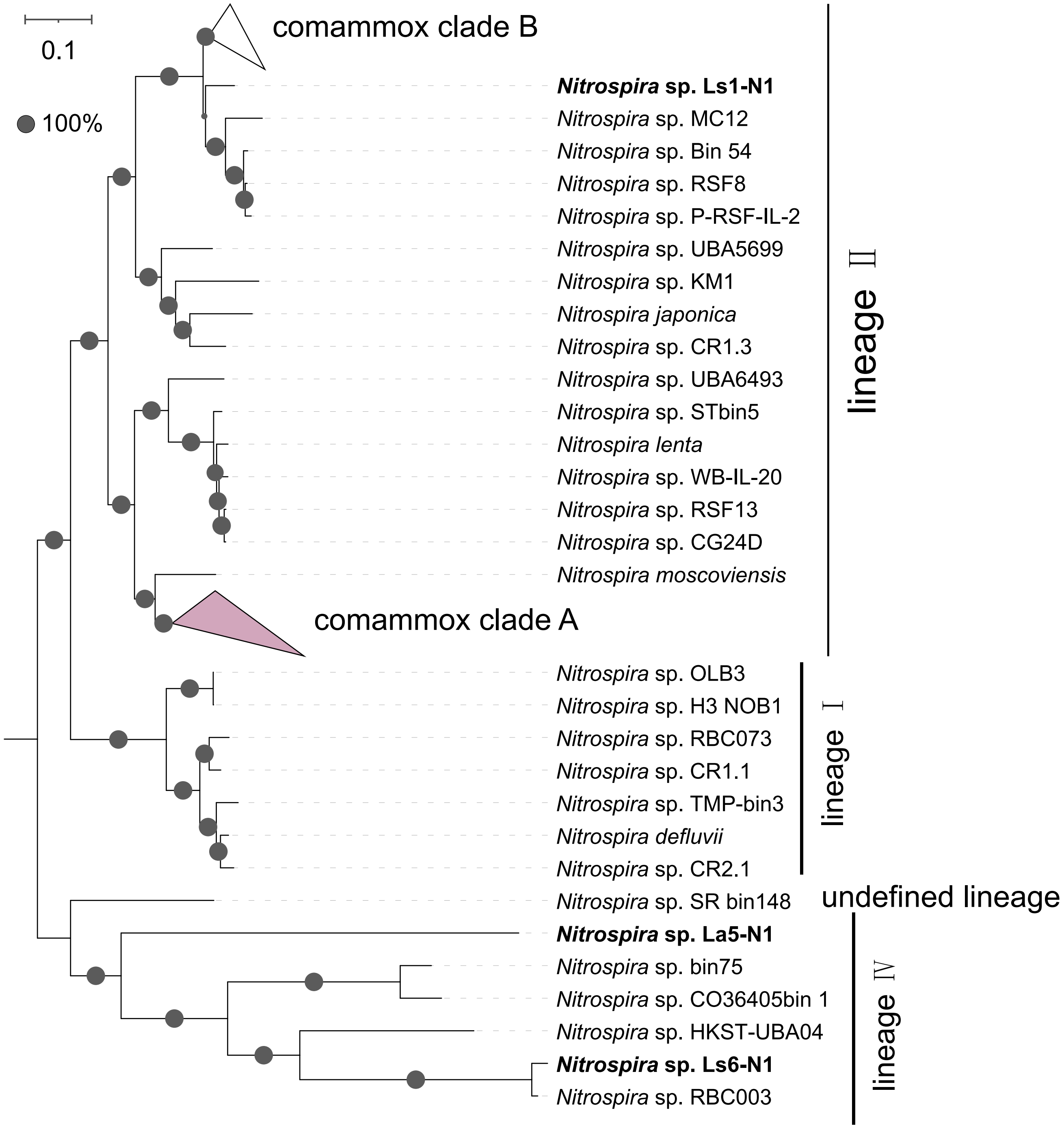

Supplementary Fig. 5. Maximum likelihood phylogenomic tree for strictly nitrite-oxidizing *Nitrospira*. The *Nitrospira* metagenome-assembled genomes (MAGs) identified in this study are emphasized in bold. The genomes of comammox *Nitrospira* are represented as collapsed branches in the tree.

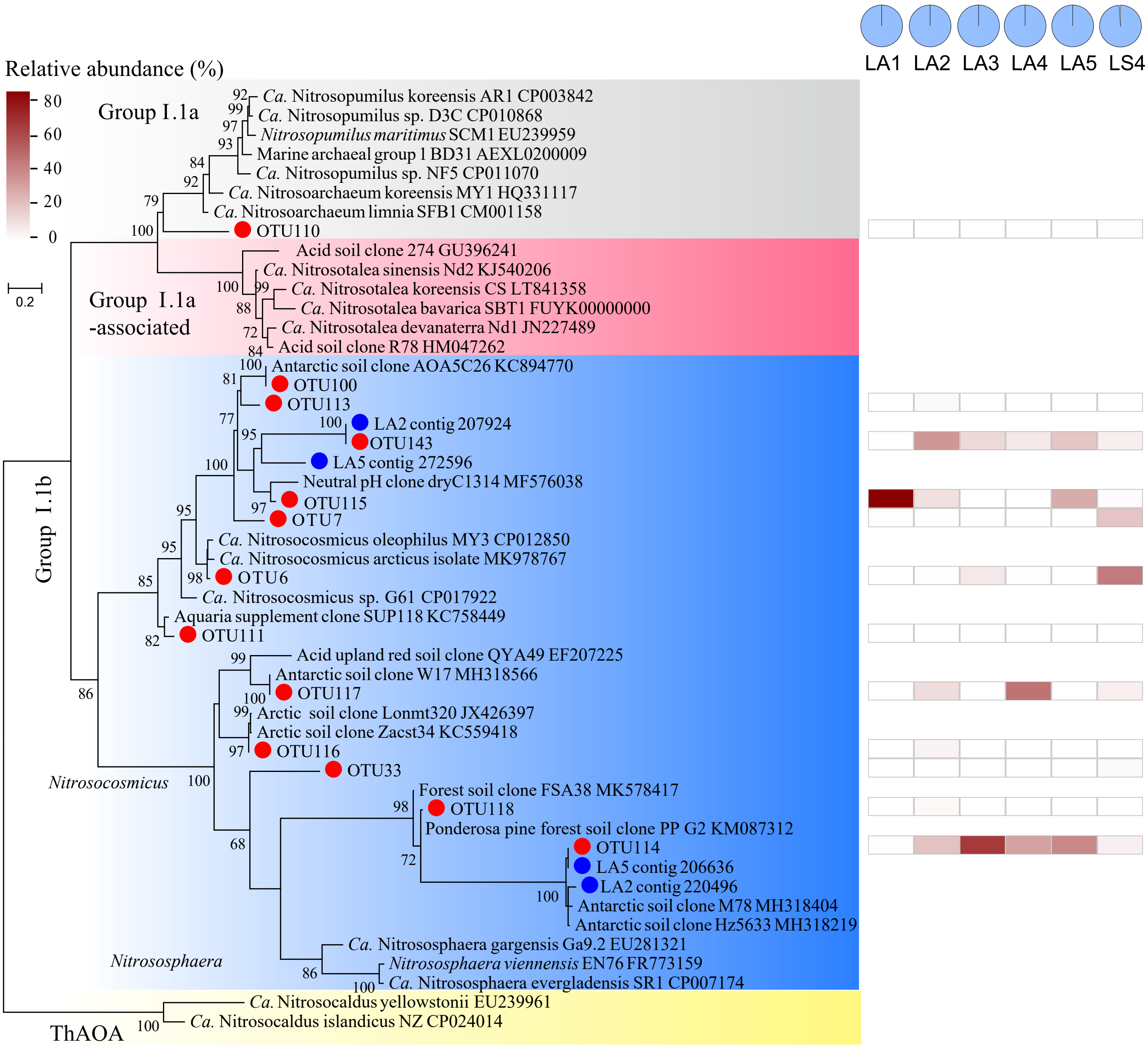

Supplementary Fig. 6. Phylogenetic analysis of AOA *amoA* gene sequences. Maximum likelihood phylogenetic tree (left) and the amplicon sequencing-based relative abundance (right) of AOA *amoA* gene sequences in LA1-LA5 (lake sediments) and LS4 (soil) from Larsemann Hills, East Antarctica. The pie charts in the top right corner represent the relative abundance patterns of different AOA groups, marked with the same colors as those in the tree background. The representative OTU sequences obtained by amplicon and metagenomic sequencing are denoted by red and blue circles, respectively.

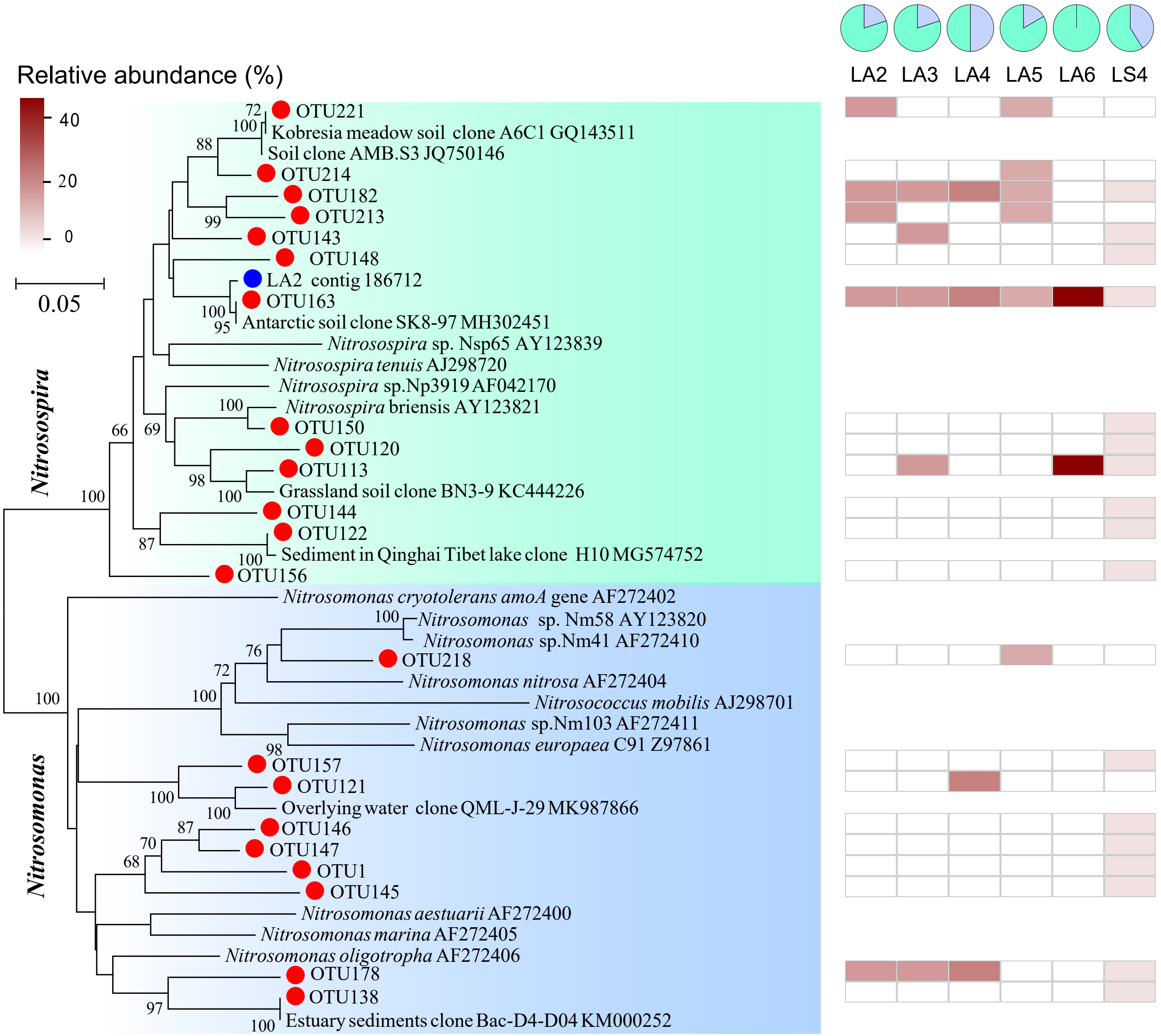

Supplementary Fig. 7. Phylogenetic analysis of AOB *amoA* gene sequences. Maximum likelihood phylogenetic tree (left) and amplicon sequencing-based relative abundance (right) of AOB *amoA* gene sequences in LA2-LA6 (lake sediments) and LS4 (soil) from Larsemann Hills, East Antarctica. The pie charts in the top right corner display the relative abundance patterns of various AOB genera, with the same colors as used in the tree background. The representative OTU sequences obtained by amplicon and metagenomic sequencing are denoted by red and blue circles, respectively.

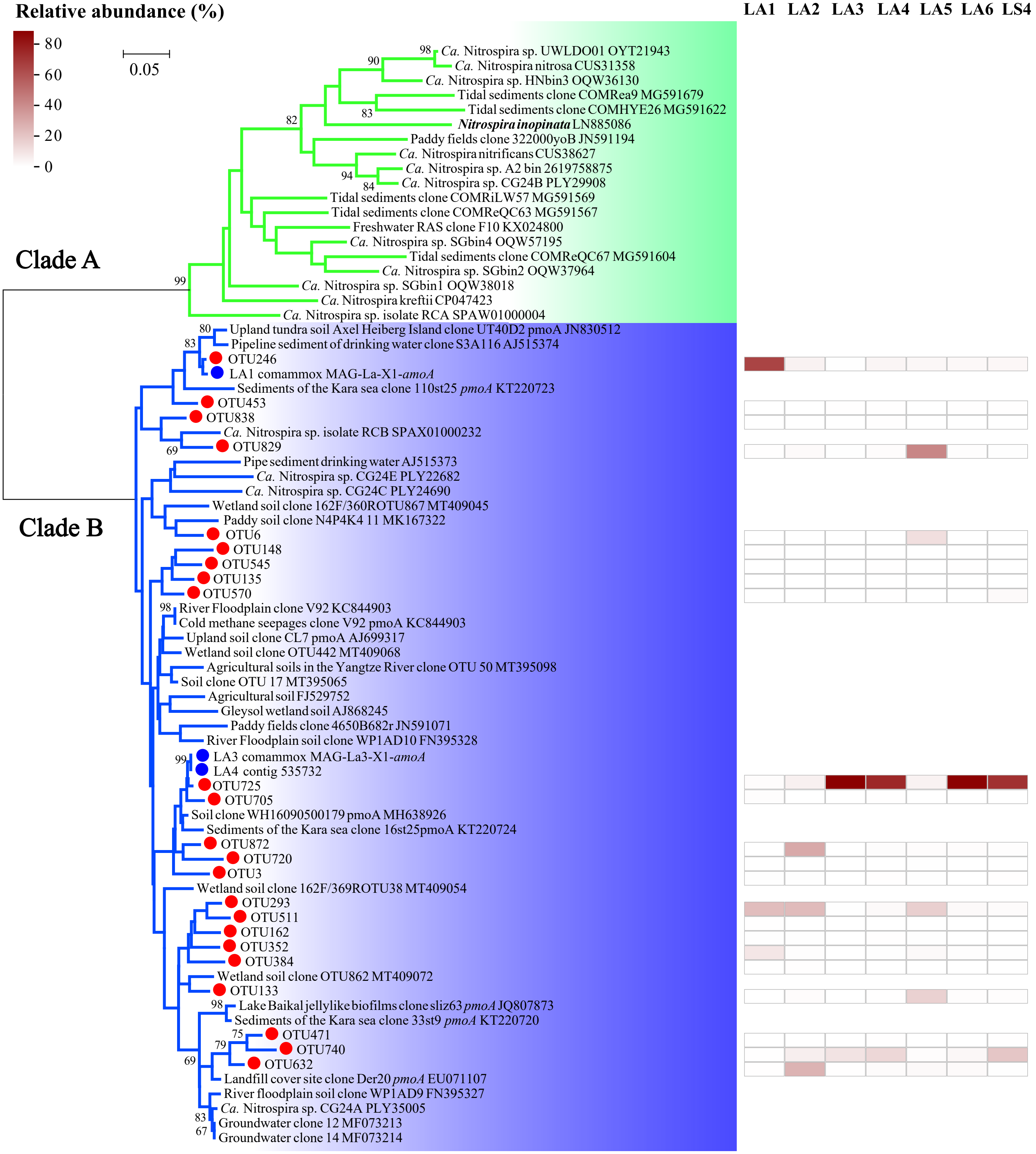

Supplementary Fig. 8. Phylogenetic analysis of comammox *Nitrospira amoA* gene sequences. Maximum likelihood phylogenetic tree (left) and amplicon sequencing-based relative abundance (right) of comammox *Nitrospira amoA* sequences in LA1-LA6 (lake sediments) and LS4 (soil) from Larsemann Hills, East Antarctica. The representative OTU sequences obtained by amplicon and metagenomic sequencing are denoted by red and blue circles, respectively.

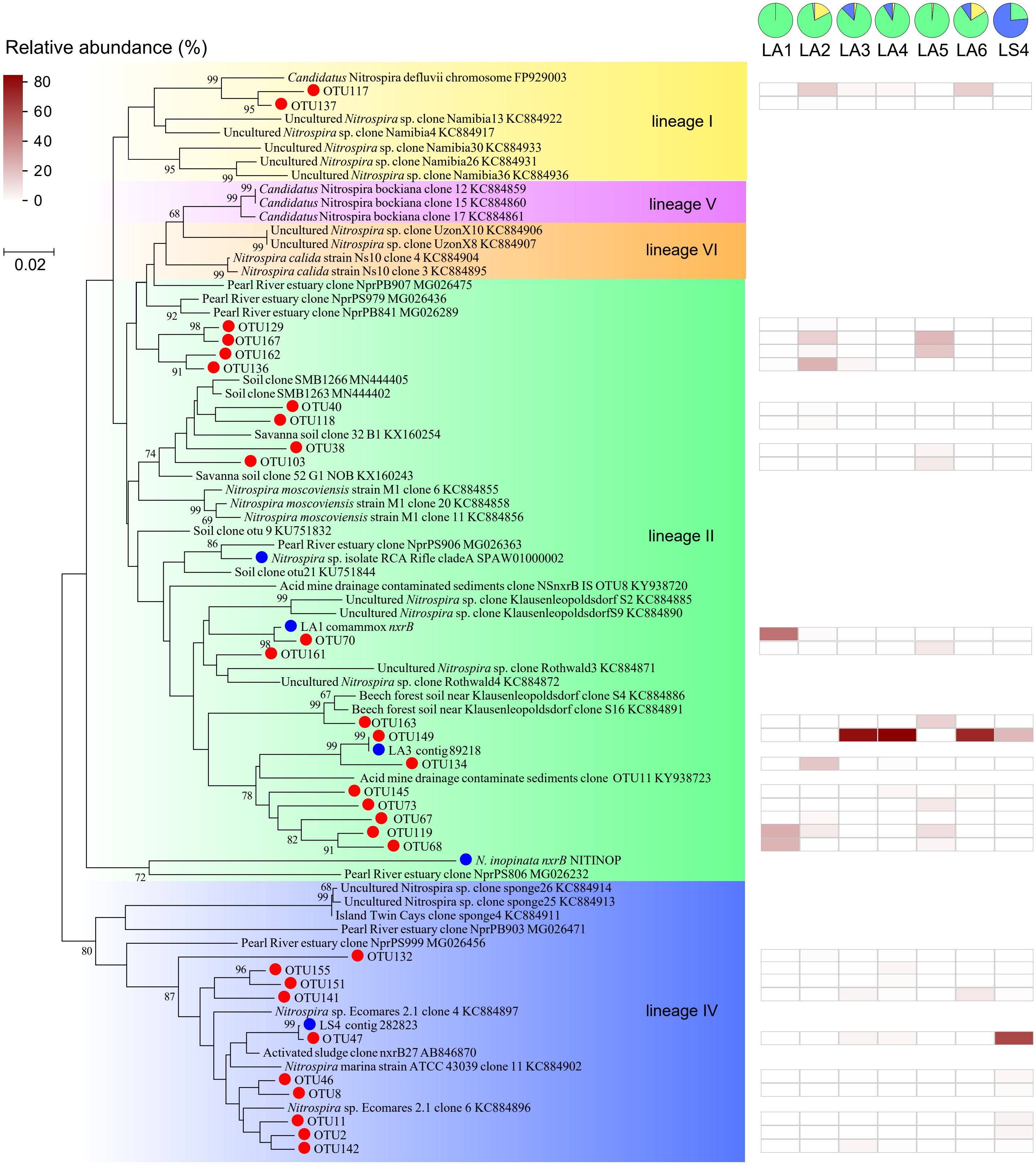

Supplementary Fig. 9. Phylogenetic analysis of *Nitrospira nxrB* gene sequences. Maximum likelihood phylogenetic tree (left) and the amplicon sequencing-based relative abundance (right) of *Nitrospira nxrB* gene sequences in LA1-LA6 (lake sediments) and LS4 (soil) from Larsemann Hills, East Antarctica. The pie charts in the top right corner indicate the relative abundance patterns of different *Nitrospira* lineages, using the same colors as those in the tree background. The representative OTU sequences obtained by amplicon and metagenomic sequencing are denoted by red and blue circles, respectively.

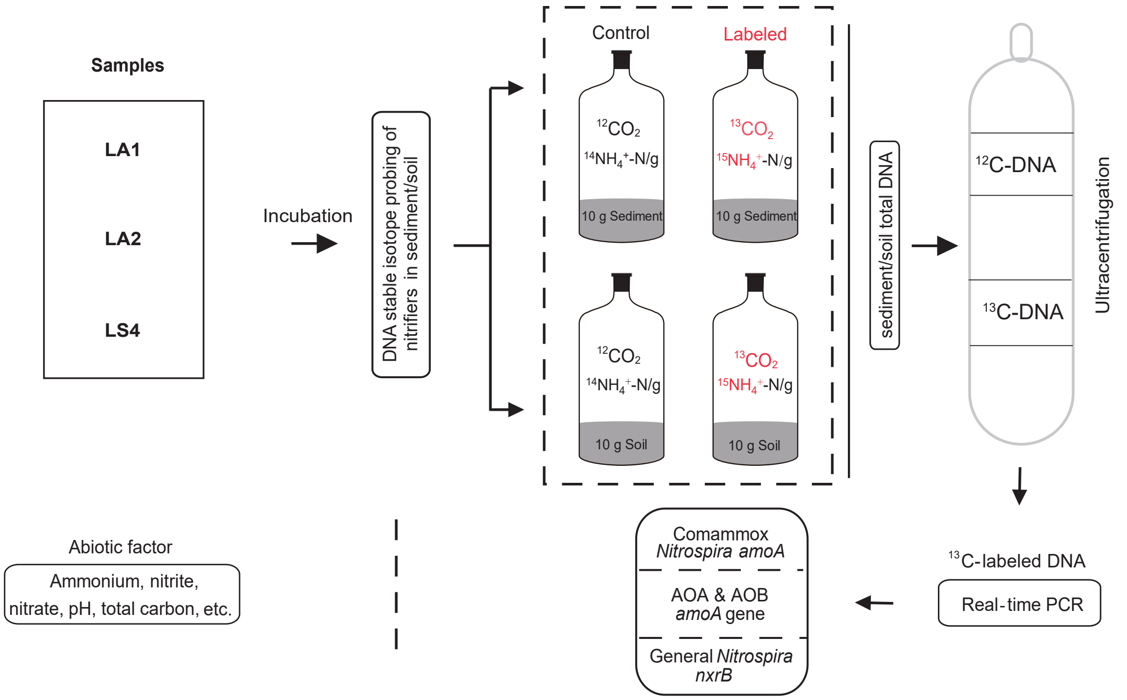

Supplementary Fig. 10. Flow chart detailing the DNA-SIP microcosm experiment. The incubations were conducted on two lake sediment samples (LA1 and LA2) and one soil sample (LS4) from Larsemann Hills, East Antarctica.

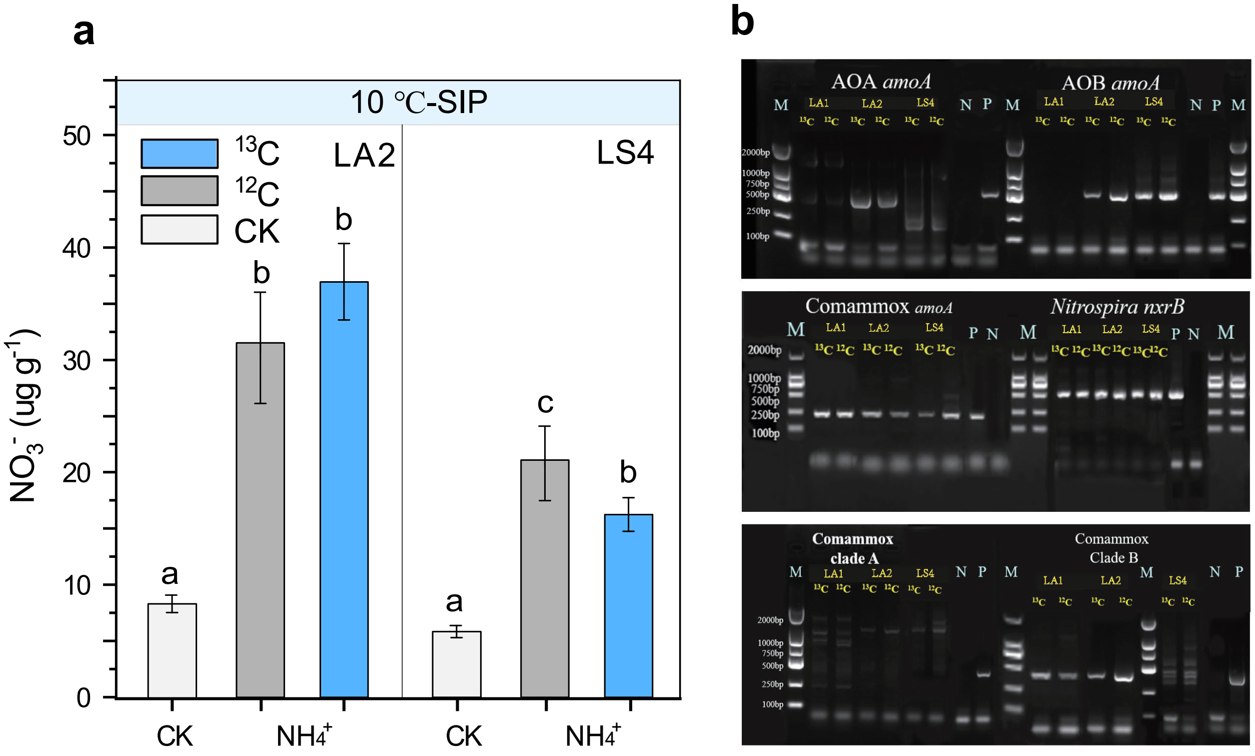

Supplementary Fig. 11. Nitrate production and the active nitrifying groups identified from the DNA-SIP microcosm incubations. (a) Accumulated nitrate concentrations in samples LA2 and LS4 following a 56-day DNA-SIP incubation at a temperature of 10 °C, using ammonium as the substrate. The nitrate for LA1 is show in Fig. 4. **(b)** Agarose gel electrophoresis results of the PCR products for AOA, AOB, comammox *amoA*, and *Nitrospira nxrB* genes in DNA extracted from the LA1 and LA2 sediments and LS4 soil after 10 °C-SIP microcosm incubations.

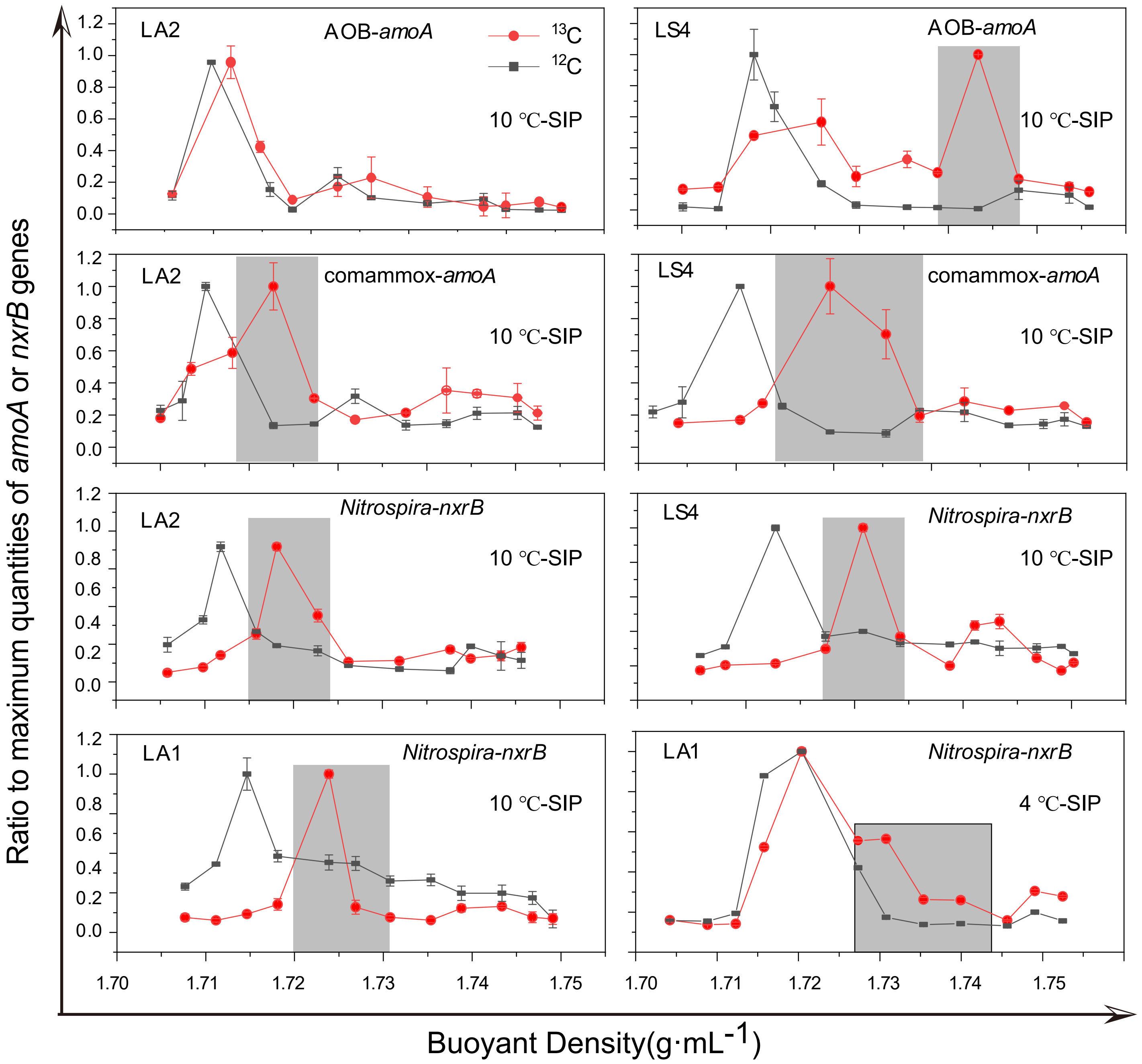

Supplementary Fig. 12. Evidence of activity of AOB, comammox *Nitrospira* and total *Nitrospira*. qPCR-derived quantitative distribution and relative abundance of AOB and comammox *Nitrospira amoA* and *Nitrospira nxrB* genes in the DNA-SIP density fractions obtained from $^{13}CO_2$ and $^{12}CO_2$-treated microcosms containing lake sediment from LA2 and soil from LS4 after a 56-day or 84-day incubation at 10 °C or 4 °C, respectively. The outcome for *Nitrospira nxrB* in LA1 is also included (see bottom), while the labeling for the corresponding comammox *Nitrospira amoA* is depicted in Fig. 4. Error bars indicate standard errors (n = 3).

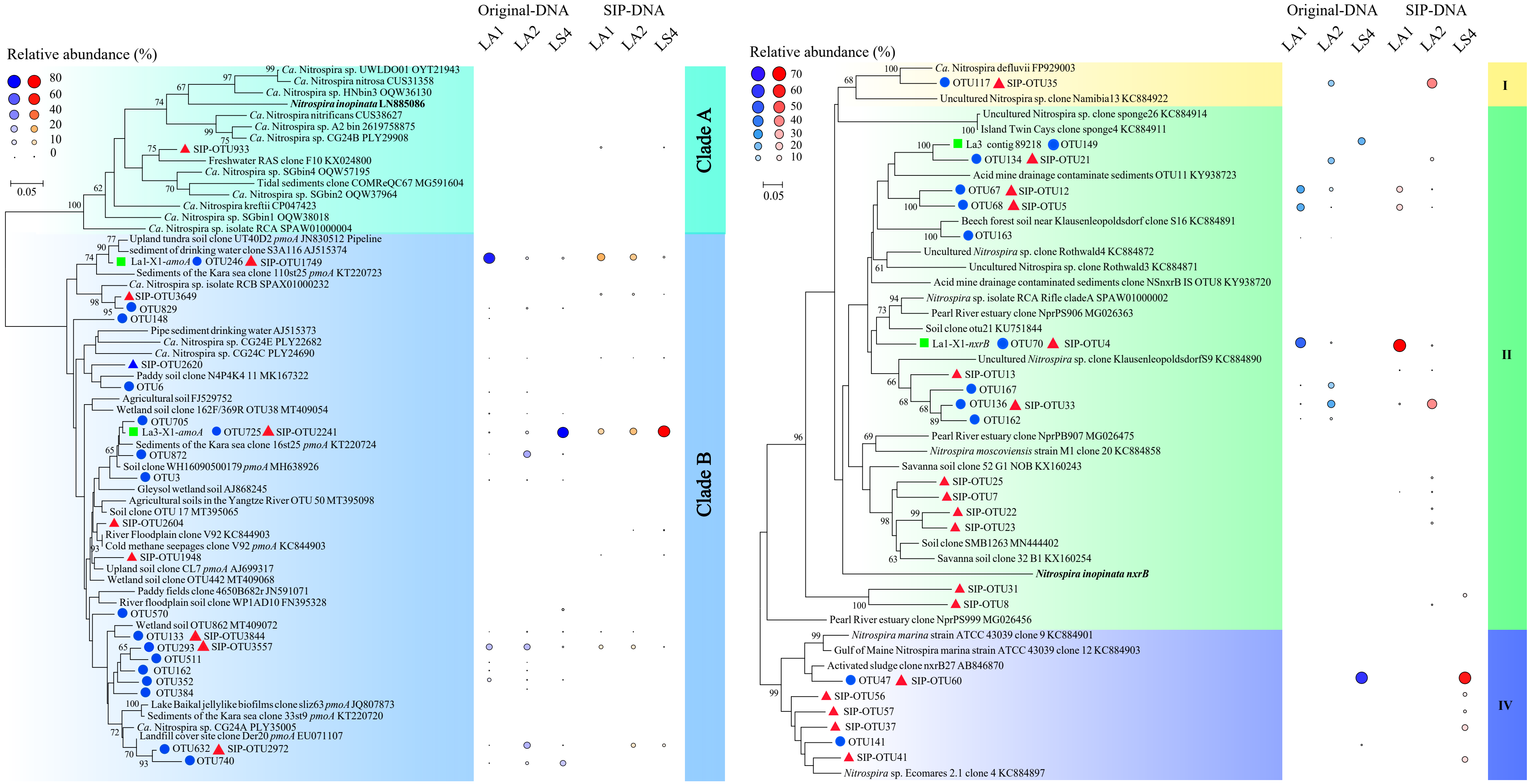

Supplementary Fig. 13. **Comparison of the diversity and relative abundance of *Nitrospira* from the original sample and 10 °C incubation SIP-DNA.** Maximum-likelihood phylogenetic trees and amplicon sequencing-derived relative abundances of OTUs in original sample DNA (red) and heavy-fraction DNA (blue) from $^{13}CO_2$-SIP incubations of **(a)** comammox *Nitrospira amoA* gene sequences and **(b)** *Nitrospira*-affiliated *nxrB* gene sequences. *AmoA* and *nxrB* genes retrieved from the metagenome-derived contigs and MAGs in this study are indicated in green.

Supplementary References

1. Pjevac, P.*, et al.* AmoA-targeted polymerase chain reaction primers for the specific detection and quantification of comammox *Nitrospira* in the environment. *Front. Microbiol.* **8**, 1508 (2017).

2. Alves, R. J.*, et al.* Nitrification rates in Arctic soils are associated with functionally distinct populations of ammonia-oxidizing archaea. *ISME J.* **7**, 1620-1631 (2013).

3. Hayashi, K.*, et al.* Ammonia oxidation potentials and ammonia oxidizers of Lichen-Moss vegetatedsoils at two ice-free areas in East Antarctica. *Microbes. Environ.* **35**, (2020).

4. Voytek, M. A., Priscu, J. C., Ward, B. B. The distribution and relative abundance of ammonia-oxidizing bacteria in lakes of the McMurdo Dry Valley, Antarctica. *Hydrobiologia* **401**, 113-130 (1999).

5. Daebeler, A.*, et al.* Exploring the upper pH limits of nitrite oxidation: diversity, ecophysiology, and adaptive traits of haloalkalitolerant *Nitrospira*. *ISME J.* **14**, 2967-2979 (2020).

6. Ortiz, M.*, et al.* Multiple energy sources and metabolic strategies sustain microbial diversity in Antarctic desert soils. *Proc. Natl. Acad. Sci. USA* **118**, (2021).

7. Pester, M.*, et al. AmoA*-based consensus phylogeny of ammonia-oxidizing archaea and deep sequencing of *amoA* genes from soils of four different geographic regions. *Environ. Microbiol.* **14**, 525-539 (2012).

8. Rotthauwe, J., Witzel, K., Liesack, W. The ammonia monooxygenase structural gene *amoA* as a functional marker: molecular fine-scale analysis of natural ammonia-oxidizing populations. *Appl. Environ. Microbiol.* **63**, 4704-4712 (1997).

9. *Fowler*, S. J., Palomo, A., Dechesne, A., Mines, P. D., Smets, B. F. Comammox *Nitrospira* are abundant ammonia oxidizers in diverse groundwater-fed rapid sand filter communities. *Environ. Microbiol.* **20**, 1002-1015 (2018).

10. Pester, M.*, et al.* NxrB encoding the beta subunit of nitrite oxidoreductase as functional and phylogenetic marker for nitrite-oxidizing *Nitrospira. Environ. Microbiol.* **16**, 3055-3071 (2014).

11. Bolyen, E.*, et al.* Reproducible, interactive, scalable and extensible microbiome data science using QIIME 2. *Nat. Biotechnol.* **37**, 852-857 (2019).

12. Callahan, B. J., McMurdie, P. J., Rosen, M. J., Han, A. W., Johnson, A. J., Holmes, S. P. DADA2: High-resolution sample inference from Illumina amplicon data. *Nat. Methods.* **13**, 581-583 (2016).

13. Nguyen, L. T., Schmidt, H. A., von Haeseler, A., Minh, B. Q. IQ-TREE: a fast and effective

stochastic algorithm for estimating maximum-likelihood phylogenies. *Mol. Biol. Evol.* **32**, 268-274 (2015).

14. Chen, S., Zhou, Y., Chen, Y., Gu, J. fastp: an ultra-fast all-in-one FASTQ preprocessor. *Bioinformatics* **34**, i884-i890 (2018).

15. Tu, Q., Lin, L., Cheng, L., Deng, Y., He, Z. NCycDB: a curated integrative database for fast and accurate metagenomic profiling of nitrogen cycling genes. *Bioinformatics*, (2018).

16. Eddy, S. R. Accelerated profile HMM searches. *PLoS Comput. Biol.* **7**, e1002195 (2011).

17. Capella-Gutiérrez, S., Silla-Martínez, J. M., Gabaldón, T. trimAl: a tool for automated alignment trimming in large-scale phylogenetic analyses. *Bioinformatics* **25**, 1972-1973 (2009).

18. Minh, B. Q., Nguyen, M. A., von Haeseler, A. Ultrafast approximation for phylogenetic bootstrap. *Mol. Biol. Evol.* **30**, 1188-1195 (2013).

19. Wang, B., Zhao, J., Guo, Z., Ma, J., Xu, H., Jia, Z. Differential contributions of ammonia oxidizers and nitrite oxidizers to nitrification in four paddy soils. *ISME J.* **9**, 1062-1075 (2015).

20. Jia, Z. J., Conrad, R. Bacteria rather than Archaea dominate microbial ammonia oxidation in an agricultural soil. *Environ. Microbiol.* **11**, 1658-1671 (2009).

21. Pester, M., *et al. amoA*-based consensus phylogeny of ammonia-oxidizing archaea and deep sequencing of *amoA* genes from soils of four different geographic regions. *Environ. Microbiol.* **14**, 525-539 (2012).

22. Yang, Y., *et al.* Specific and effective detection of anammox bacteria using PCR primers targeting the 16S rRNA gene and functional genes. *Sci. Total Environ.* **734**, 139387 (2020).

23. Pester, M., *et al. NxrB* encoding the beta subunit of nitrite oxidoreductase as functional and phylogenetic marker for nitrite-oxidizing *Nitrospira*. *Environ. Microbiol.* **16**, 3055-3071 (2014).

24. Wang, Y., *et al.* Co-occurrence and distribution of nitrite-dependent anaerobic ammonium and methane-oxidizing bacteria in a paddy soil. *FEMS Microbiol. Lett.* **336**, 79-88 (2012).

25. Allen, A. E., Booth, M. G., Frischer, M. E., Verity, P. G., Zehr, J. P., Zani, S. Diversity and detection of nitrate assimilation genes in marine bacteria. *Appl. Environ. Microbiol.* **67**, 5343-5348 (2001).

26. Alcantara-Hernandez, R. J., Valenzuela-Encinas, C., Zavala-Diaz de la Serna, F. J., Rodriguez-Revilla, J., Dendooven, L., Marsch, R. Haloarchaeal assimilatory nitrate-reducing communities from a saline alkaline soil. *FEMS Microbiol. Lett.* **298**, 56-66 (2009).

27. Schaedel, M., *et al.* Temporal assessment of N-cycle microbial functions in a tropical agricultural

soil using gene co-occurrence networks. *PLoS One* **18**, e0281442 (2023).

28. Mohan, S. B., Schmid, M., Jetten, M., Cole, J. Detection and widespread distribution of the *nrfA* gene encoding nitrite reduction to ammonia, a short circuit in the biological nitrogen cycle that competes with denitrification. *FEMS Microbiol. Ecol.* **49**, 433-443 (2004).

29. Throback, I. N., Enwall, K., Jarvis, A., Hallin, S. Reassessing PCR primers targeting *nirS*, *nirK* and *nosZ* genes for community surveys of denitrifying bacteria with DGGE. *FEMS Microbiol. Ecol.* **49**, 401-417 (2004).

30. Henry, S., Baudoin, E., Lopez-Gutierrez, J. C., Martin-Laurent, F., Brauman, A., Philippot, L. Quantification of denitrifying bacteria in soils by *nirK* gene targeted real-time PCR. *J. Microbiol. Methods.* **59**, 327-335 (2004).

31. Jones, C. M., Graf, D. R., Bru, D., Philippot, L., Hallin, S. The unaccounted yet abundant nitrous oxide-reducing microbial community: a potential nitrous oxide sink. *ISME J.* **7**, 417-426 (2013).

32. Wang, C., *et al.* Impact of 25 years of inorganic fertilization on diazotrophic abundance and community structure in an acidic soil in southern China. *Soil Biol. Biochem.* **113**, 240-249 (2017).

33. Flanagan, D. A., Gregory, L. G., Carter, J. P., Karakas-Sen, A., Richardson, D. J., Spiro, S. Detection of genes for periplasmic nitrate reductase in nitrate respiring bacteria and in community DNA. *FEMS Microbiol. Lett.* **177**, 263-270 (1999).

Appendix 1: Code for Analysis of AOA-*amoA* Gene Sequences Using QIIME2

```
PATH=/opt/biosoft/miniconda3_for_QIIME2/bin:$PATH
source activate qiime2-2020.6

## sample-metadata.tsv
##sample-id        forward-barcodes        reverse-barcodes
Lin027   GATCTGCA        CTACGATG
Lin028   GATCTGCA        GACATAGC
Lin029   GATCTGCA        GATCTGCA
Lin032   GATCTGCA        GCGTATGA
Lin033   GATCTGCA        GTATGCGA

mkdir muxed-pe-barcode-in-seq (forward.fastq.gz reverse.fastq.gz)

qiime tools import    --type MultiplexedPairedEndBarcodeInSequence    \
    --input-path muxed-pe-barcode-in-seq    \
    --output-path multiplexed-seqs.qza
------------------------------------

## qiime cutadapt
qiime cutadapt demux-paired --i-seqs multiplexed-seqs.qza \
    --m-forward-barcodes-file sample-metadata.tsv \
    --m-forward-barcodes-column forward-barcodes \
    --m-reverse-barcodes-file sample-metadata.tsv \
    --m-reverse-barcodes-column reverse-barcodes \
    --o-per-sample-sequences per_sample_sequences.qza \
    --o-untrimmed-sequences untrimmed_sequences.qza

##per_sample_sequences.qza
qiime cutadapt demux-paired --i-seqs multiplexed-seqs.qza \
    --m-forward-barcodes-file sample-metadata.tsv \
    --m-forward-barcodes-column forward-barcodes \
    --o-per-sample-sequences per_sample_sequences.qza \
    --o-untrimmed-sequences untrimmed_sequences.qza

## data2
qiime dada2 denoise-paired \
--i-demultiplexed-seqs   per_sample_sequences.qza \
--p-trim-left-f 0 \
--p-trim-left-r 0 \
```

```
--p-trunc-len-f 0 \
--p-trunc-len-r 0 \
--o-table table.qza \
--o-representative-sequences rep-seqs.qza \
--o-denoising-stats denoising-stats-data.qza \
--verbose \
--p-n-threads 0

time qiime vsearch cluster-features-de-novo \
--i-table table.qza \
--i-sequences rep-seqs.qza \
--p-perc-identity 0.95 \
--o-clustered-table table-dn-95.qza \
--o-clustered-sequences rep-seqs-dn-95.qza

time qiime vsearch cluster-features-open-reference \
--i-table table.qza \
--i-sequences rep-seqs.qza \
--i-reference-sequences AOA_amoA.qza \
--p-perc-identity 0.95 \
--o-clustered-table table-or-95.qza \
--o-clustered-sequences rep-seqs-or-95.qza \
--o-new-reference-sequences new-ref-seqs-or-95.qza

time qiime tools export --input-path table-dn-95.qza --output-path exported-tabledn95
time qiime tools export --input-path rep-seqs-dn-95.qza --output-path sequencednfas95
biom convert -i exported-tabledn95/feature-table.biom   -o exported-tabledn95/feature-table.txt --
to-tsv
```

Appendix 2: Code for Analysis of AOB-*amoA* Gene Sequences Using QIIME2

```
PATH=/opt/biosoft/miniconda3_for_QIIME2/bin:$PATH
source activate qiime2-2020.6

## sample-metadata.tsv
##sample-id	forward-barcodes	reverse-barcodes
Lin027	GATCTGCA	CTACGATG
Lin028	GATCTGCA	GACATAGC
Lin029	GATCTGCA	GATCTGCA
Lin032	GATCTGCA	GCGTATGA
Lin033	GATCTGCA	GTATGCGA

mkdir muxed-pe-barcode-in-seq (forward.fastq.gz reverse.fastq.gz)

qiime tools import	--type MultiplexedPairedEndBarcodeInSequence	\
	--input-path muxed-pe-barcode-in-seq	\
	--output-path multiplexed-seqs.qza
------------------------------------

## qiime cutadapt
qiime cutadapt demux-paired --i-seqs multiplexed-seqs.qza \
	--m-forward-barcodes-file sample-metadata.tsv \
	--m-forward-barcodes-column forward-barcodes \
	--m-reverse-barcodes-file sample-metadata.tsv \
	--m-reverse-barcodes-column reverse-barcodes \
	--o-per-sample-sequences per_sample_sequences.qza \
	--o-untrimmed-sequences untrimmed_sequences.qza

##per_sample_sequences.qza
qiime cutadapt demux-paired --i-seqs multiplexed-seqs.qza \
	--m-forward-barcodes-file sample-metadata.tsv \
	--m-forward-barcodes-column forward-barcodes \
	--o-per-sample-sequences per_sample_sequences.qza \
	--o-untrimmed-sequences untrimmed_sequences.qza

## data2
qiime dada2 denoise-paired \
--i-demultiplexed-seqs	per_sample_sequences.qza \
--p-trim-left-f 0 \
--p-trim-left-r 0 \
```

```
--p-trunc-len-f 0 \
--p-trunc-len-r 0 \
--o-table table.qza \
--o-representative-sequences rep-seqs.qza \
--o-denoising-stats denoising-stats-data.qza \
--verbose \
--p-n-threads 0

time qiime vsearch cluster-features-de-novo \
--i-table table.qza \
--i-sequences rep-seqs.qza \
--p-perc-identity 0.95 \
--o-clustered-table table-dn-95.qza \
--o-clustered-sequences rep-seqs-dn-95.qza

time qiime vsearch cluster-features-open-reference \
--i-table table.qza \
--i-sequences rep-seqs.qza \
--i-reference-sequences AOB_amoA.qza \
--p-perc-identity 0.95 \
--o-clustered-table table-or-95.qza \
--o-clustered-sequences rep-seqs-or-95.qza \
--o-new-reference-sequences new-ref-seqs-or-95.qza

time qiime tools export --input-path table-dn-95.qza --output-path exported-tabledn95
time qiime tools export --input-path rep-seqs-dn-95.qza --output-path sequencednfas95
biom convert -i exported-tabledn95/feature-table.biom   -o exported-tabledn95/feature-table.txt --
to-tsv
```

Appendix 3: Code for Analysis of comammox *Nitrospira-amoA* Gene Sequences Using QIIME2

```
PATH=/opt/biosoft/miniconda3_for_QIIME2/bin:$PATH
source activate qiime2-2020.6

## sample-metadata.tsv
##sample-id            forward-barcodes              reverse-barcodes
Lin027    GATCTGCA              CTACGATG
Lin028    GATCTGCA              GACATAGC
Lin029    GATCTGCA              GATCTGCA
Lin032    GATCTGCA              GCGTATGA
Lin033    GATCTGCA              GTATGCGA

mkdir muxed-pe-barcode-in-seq (forward.fastq.gz reverse.fastq.gz)

qiime tools import      --type MultiplexedPairedEndBarcodeInSequence      \
      --input-path muxed-pe-barcode-in-seq      \
      --output-path multiplexed-seqs.qza
------------------------------------

## qiime cutadapt
qiime cutadapt demux-paired --i-seqs multiplexed-seqs.qza \
      --m-forward-barcodes-file sample-metadata.tsv \
      --m-forward-barcodes-column forward-barcodes \
      --m-reverse-barcodes-file sample-metadata.tsv \
      --m-reverse-barcodes-column reverse-barcodes \
      --o-per-sample-sequences per_sample_sequences.qza \
      --o-untrimmed-sequences untrimmed_sequences.qza

##per_sample_sequences.qza
qiime cutadapt demux-paired --i-seqs multiplexed-seqs.qza \
      --m-forward-barcodes-file sample-metadata.tsv \
      --m-forward-barcodes-column forward-barcodes \
      --o-per-sample-sequences per_sample_sequences.qza \
      --o-untrimmed-sequences untrimmed_sequences.qza

## data2
qiime dada2 denoise-paired \
--i-demultiplexed-seqs    per_sample_sequences.qza \
--p-trim-left-f 0 \
```

```
--p-trim-left-r 0 \
--p-trunc-len-f 0 \
--p-trunc-len-r 0 \
--o-table table.qza \
--o-representative-sequences rep-seqs.qza \
--o-denoising-stats denoising-stats-data.qza \
--verbose \
--p-n-threads 0

time qiime vsearch cluster-features-de-novo \
--i-table table.qza \
--i-sequences rep-seqs.qza \
--p-perc-identity 0.95 \
--o-clustered-table table-dn-95.qza \
--o-clustered-sequences rep-seqs-dn-95.qza

time qiime vsearch cluster-features-open-reference \
--i-table table.qza \
--i-sequences rep-seqs.qza \
--i-reference-sequences comammox_amoA.qza \
--p-perc-identity 0.95 \
--o-clustered-table table-or-95.qza \
--o-clustered-sequences rep-seqs-or-95.qza \
--o-new-reference-sequences new-ref-seqs-or-95.qza

time qiime tools export --input-path table-or-95.qza --output-path exported-tabledn95
time qiime tools export --input-path rep-seqs-or-95.qza --output-path sequencednfas95
biom convert -i exported-tableor95/feature-table.biom  -o exported-tabledn95/feature-table.txt --
to-tsv
```

Appendix 4: Code for Analysis of *Nitrospira-nxrB* Gene Sequences Using QIIME2

```
PATH=/opt/biosoft/miniconda3_for_QIIME2/bin:$PATH
source activate qiime2-2020.6

## sample-metadata.tsv
##sample-id        forward-barcodes        reverse-barcodes
Lin027   GATCTGCA        CTACGATG
Lin028   GATCTGCA        GACATAGC
Lin029   GATCTGCA        GATCTGCA
Lin032   GATCTGCA        GCGTATGA
Lin033   GATCTGCA        GTATGCGA

mkdir muxed-pe-barcode-in-seq (forward.fastq.gz reverse.fastq.gz)

qiime tools import    --type MultiplexedPairedEndBarcodeInSequence    \
    --input-path muxed-pe-barcode-in-seq    \
    --output-path multiplexed-seqs.qza
-----------------------------------

## qiime cutadapt
qiime cutadapt demux-paired --i-seqs multiplexed-seqs.qza \
    --m-forward-barcodes-file sample-metadata.tsv \
    --m-forward-barcodes-column forward-barcodes \
    --m-reverse-barcodes-file sample-metadata.tsv \
    --m-reverse-barcodes-column reverse-barcodes \
    --o-per-sample-sequences per_sample_sequences.qza \
    --o-untrimmed-sequences untrimmed_sequences.qza

##per_sample_sequences.qza
qiime cutadapt demux-paired --i-seqs multiplexed-seqs.qza \
    --m-forward-barcodes-file sample-metadata.tsv \
    --m-forward-barcodes-column forward-barcodes \
    --o-per-sample-sequences per_sample_sequences.qza \
    --o-untrimmed-sequences untrimmed_sequences.qza

## data2
qiime dada2 denoise-paired \
--i-demultiplexed-seqs   per_sample_sequences.qza \
--p-trim-left-f 0 \
--p-trim-left-r 0 \
```

```
--p-trunc-len-f 0 \
--p-trunc-len-r 0 \
--o-table table.qza \
--o-representative-sequences rep-seqs.qza \
--o-denoising-stats denoising-stats-data.qza \
--verbose \
--p-n-threads 0

time qiime vsearch cluster-features-de-novo \
--i-table table.qza \
--i-sequences rep-seqs.qza \
--p-perc-identity 0.95 \
--o-clustered-table table-dn-95.qza \
--o-clustered-sequences rep-seqs-dn-95.qza

time qiime vsearch cluster-features-open-reference \
--i-table table.qza \
--i-sequences rep-seqs.qza \
--i-reference-sequences NOB_nxrB.qza \
--p-perc-identity 0.95 \
--o-clustered-table table-or-95.qza \
--o-clustered-sequences rep-seqs-or-95.qza \
--o-new-reference-sequences new-ref-seqs-or-95.qza

time qiime tools export --input-path table- or -95.qza --output-path exported-tabledn95
time qiime tools export --input-path rep-seqs- or -95.qza --output-path sequencednfas95
biom convert -i exported-table or 95/feature-table.biom   -o exported-tabledn95/feature-table.txt --
to-tsv
```
