# Supplementary material for: Unveiling unique microbial nitrogen cycling and nitrification driver in coastal Antarctica
Source: Nat Commun. 2024 Apr 12;15:3143. doi: 10.1038/s41467-024-47392-4 (PMC11014942; doi:10.1038/s41467-024-47392-4)
Supplement: Supplementary file 3 — Description of Additional Supplementary File [file 41467_2024_47392_MOESM3_ESM.pdf]

Description of Additional Supplementary File

File Name: Supplementary Data 1

Description: Summary of the obtained 1968 metagenome-assembled genomes (MAGs).

File Name: Supplementary Data 2

Description: Statistics of presence or absence of key metabolic marker genes in all retrieved metagenome-assembled genomes (MAGs).
